# Primates facing climate crisis in a tropical forest hotspot will lose climatic suitable geographical range

**DOI:** 10.1038/s41598-022-26756-0

**Published:** 2023-01-12

**Authors:** Míriam Plaza Pinto, Raone Beltrão-Mendes, Maurício Talebi, Adriana Almeida de Lima

**Affiliations:** 1 Departamento de Ecologia, Centro de Biociências, Universidade Federal do Rio Grande do Norte (UFRN), 59072-970 Natal, RN Brasil; 2 Programa de Pós-Graduação em Ecologia, Centro de Biociências, Universidade Federal do Rio Grande do Norte (UFRN), 59072-970 Natal, RN Brasil; 3 Programa de Pós-Graduação em Ecologia e Conservação, Universidade Federal de Sergipe (UFS), 49100-000 São Cristóvão, SE Brasil; 4 Departamento de Ciências Ambientais, Universidade Federal de São Paulo (UNIFESP), 09972-270 Diadema, SP Brasil; 5 Programa de Pós-Graduação em Análise Ambiental Integrada, Universidade Federal de São Paulo (UNIFESP), Diadema, SP Brasil

**Keywords:** Ecology, Biodiversity, Climate-change ecology, Conservation biology

## Abstract

Global climate changes affect biodiversity and cause species distribution shifts, contractions, and expansions. Climate change and disease are emerging threats to primates, and approximately one-quarter of primates’ ranges have temperatures over historical ones. How will climate changes influence Atlantic Forest primate ranges? We used habitat suitability models and measured potential changes in area and distributions shifts. Climate change expected in 2100 may change the distribution area of Atlantic Forest primates. Fourteen species (74%) are predicted to lose more than 50% of their distribution, and nine species (47%) are predicted to lose more than 75% of their distribution. The balance was negative, indicating a potential future loss, and the strength of the reduction in the distribution is related to the severity of climate change (SSP scenarios). Directional shifts were detected to the south. The projected mean centroid latitudinal shift is ~ 51 km to the south for 2100 SSP5-8.5 scenario. The possibility of dispersal will depend on suitable routes and landscape configuration. Greenhouse gas emissions should be urgently reduced. Our results also emphasize that no more forest loss is acceptable in Atlantic Forest, and restoration, canopy bridges, friendly agroecosystems, and monitoring of infrastructure projects are urgent to enable dealing with climate change.

## Introduction

Natural or anthropic-driven climate changes affect biodiversity^1–3^. Species redistributions, for example, occur through evolutionary time due to climate changes^2,4^. Nonetheless, climate change accelerated by the human predatory use of natural resources has consequences on species abundance, demography, phenology, morphology, and geographic distributions^1,5^. In addition, ecosystem functioning and biodiversity persistence can be menaced by modifying population and species’ attributes or characteristics^6^.

The suitable climatic characteristics may change spatially due to climate change^1,3^. Intrinsic attributes, such as dispersal capacity, habitat preference, diet breadth, and generation time, will influence the species’ responses to changes in climate conditions^7–9^. Species with small body sizes and short life cycles should be favored^10^. These ecological characteristics and the intensity of climate change may influence the possibility of acclimation or adaptation to new conditions^11^, dispersal to newly suitable areas^12^, or local and even regional extinction^13,14^. Species with smaller geographic ranges are exposed to lower climatic variability, which may reflect lower ecological tolerance and more proneness to be affected by climate change^15^. On the other hand, species with large geographical ranges, are already exposed to greater climatic variability^16^ and may be more tolerant to climate changes.

Climate change can cause species geographic distribution shifts. Some species are expected to face the displacement of climatic suitability beyond their current geographic range^17^. In addition, their habitat suitability and geographic distribution may reduce^18^. In some cases, the suitability change may complement the current suitability, increasing in geographic distributions^19^. Nonetheless, natural geographic barriers or inhospitable environments may limit species redistributions^20,21^. The dispersal capacity of each species also limits the moving toward future new areas with suitable climates^22^. Species with greater dispersal capacity are predicted to reach distant areas^23,24^, which can be important in climate suitability spatial shifting. In addition, land use change, a strong anthropogenic factor, is projected to limit the dispersal capacity of some species^25^.

Patterns of species redistributions already documented are mainly related to climatic suitability, such as geographical range shifts polewards and to higher elevations^1,17,26^, or deeply in marine environments^27^. These shifts are due to climatic factors (mainly mean temperature and its seasonality) correlated with latitude and elevation^28^. The climatic suitability area along the distributions of scorpions^29^, the emblematic Brazilian pine tree *Araucaria angustifolia*^30,31^, and the excessively harvested brazilwood *Paubrasilia echinata*^32^, for example, are predicted to reduce. Additional examples include endemic birds from the Brazilian Atlantic Forest (hereafter referred to as Atlantic Forest), which had a more extensive climatic suitability distribution in the recent past^33^. Climatic suitability distribution is also predicted to reduce for Equatorian primates^34^ and reduce and shift to more elevated areas in the douc species *Pygathrix cinerea* (Vietnam)^35^. Amazonian primates are predicted to expand their climatic suitability distribution, but restrictions in dispersal may completely change the prediction, leading to a reduction^36^. The change in climatic areas suitable for baboons, genus *Papio*, is predicted to be heterogeneous among species^37^. Complex multi-directional changes in climatic suitability are predicted for Australian birds^28^. However, the prediction of latitudinal spatial changes can underestimate distributional spatial shifts to east and west^38^.

Anthropic pressures, such as logging, hunting, and agriculture, contribute to the threatened status of 65.5% of primate species^39^. According to these authors, the recent literature mentions climate change among the emerging threats to primates. Approximately one-quarter of all primate species ranges had temperatures over pre-industrial seasonal maximum temperatures, suggesting the emergence of climatic conditions outside the historical ones^40^. Primates and Eulipotyphla (moles) are the mammals that will suffer more significant losses in climatic suitability distributions due to the climate change speed and dispersal capacity^9^. In this context, Brazil is a priority country for primate conservation, with 102 species, of which 39% are threatened^41^. Twenty-six primate species occur in the Atlantic Forest, of which 19 are endemic^42,43^. The biome is considered a *hotspot* of primate vulnerability to climate change in a global evaluation that combined the number of threatened species and the severity of climate changes^42,43^. The main changes in climate expected for this biome are higher temperature, lower precipitation in the center and north regions, and higher precipitation in the south region^43^.

Some Atlantic Forest primates will face a reduction in the climatic suitability along their distribution, such as *Callicebus* and *Leontopithecus*^20,44^, and the climatic suitability area of some marmoset species (*Callithrix*) are predicted to expand while others to reduce^45^. Redistributions due to climate change are predicted to lead to changes in the spatial diversity of Atlantic Forest primates, such as reducing species richness in some parts of the biome and increasing community heterogeneity in space in the future^46^. Although there are some studies for individual species, previous research has yet to evaluate the potential redistribution and shifts in the ranges of all the primates in the Atlantic Forest imperiled biome. Here we investigated how climate changes will influence Atlantic Forest primate ranges. We expect that: 1) species’ climatic suitability distribution will change (i.e., reducing, expanding, shifting) once climate act on limiting distributions, temperature and precipitation are going to change in this biome, and previous research with some species predicted shifts; 2) the larger the current IUCN geographical ranges, the smaller will be proportional changes expected in the future climatic suitability distributions, once species with larger ranges are subject to more varied environmental conditions, and smaller range ones may have lower ecological tolerances; 3) the main trend in climatic suitability distribution direction shifts will be to higher latitudes (to the south, in the south hemisphere), following the pattern already detected for other taxonomic groups in different regions.

## Results

### Changes in distribution areas

**Figure 1:**
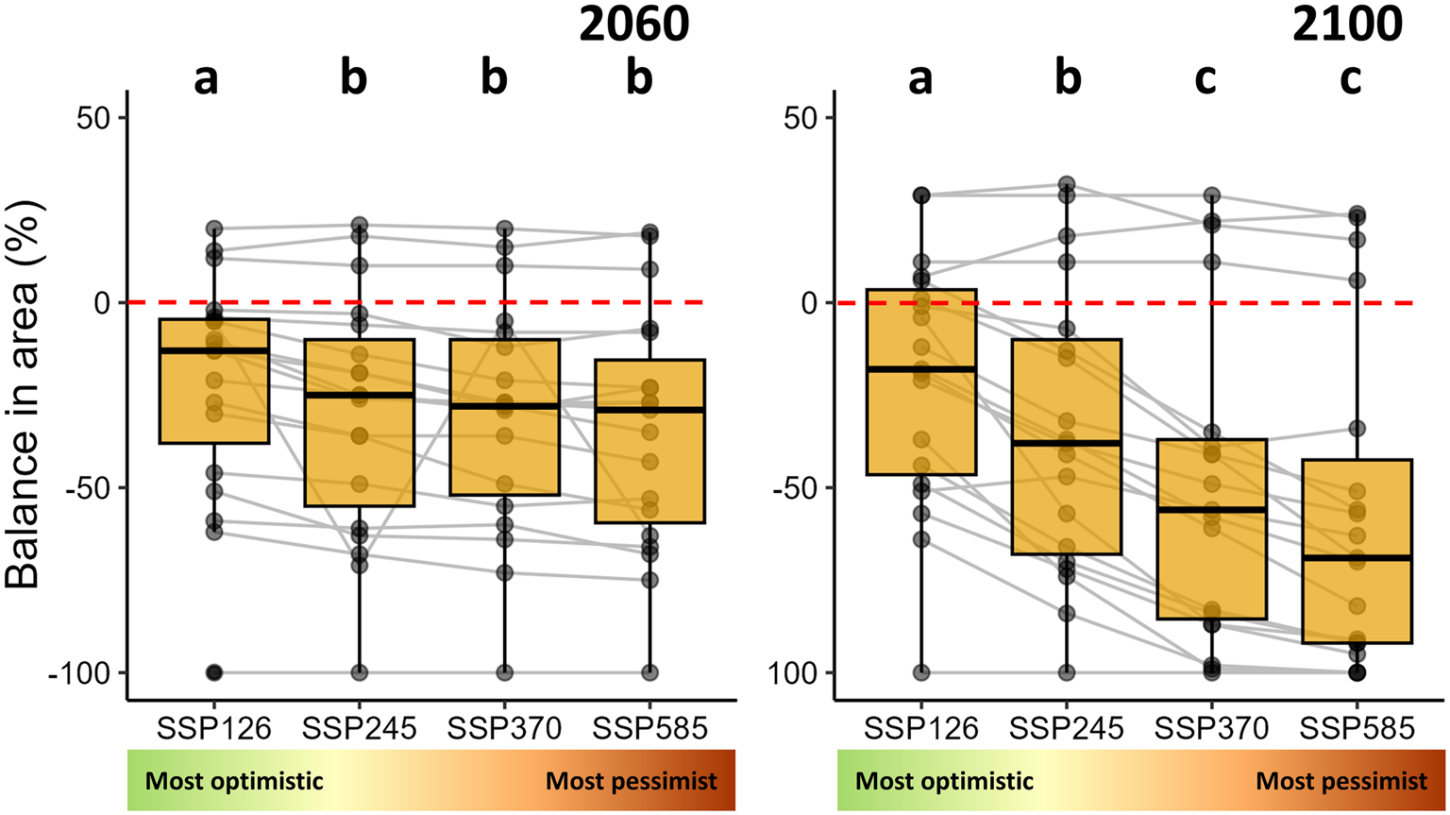
Balance in climatic suitability distribution area for Atlantic Forest endemic primate species (n = 19) in different *Shared Socioeconomic Pathways* (SSP1-2.6, the most optimistic one, SSP2-4.5, SSP3-7.0 and SSP5-8.5, the most pessimist one) for two times in the future (2060 and 2100) without considering potential dispersal. Balance (%) is the difference between area gain and area loss predicted and is proportional to the current climatic suitability distribution area. Negative values indicate a reduction, and positive values indicate a gain in distribution size predicted for the future. Medians, quartiles, and deciles are presented in boxplots. Post hoc results are represented by small letters (see detailed post hoc in Supplementary Table S2).

Climate changes expected to occur in 2100 may change the distribution area of all Atlantic Forest primate species. All these species are expected to lose some part of their climatic suitability distribution in the scenario SSP5-8.5. In this scenario, fourteen species (74%) are predicted to lose more than 50% of their current climatic suitability distribution; among those, nine species (47%) are predicted to lose more than 75% of their current climatic suitability distribution. These species are predicted to have no area gain in the climatic suitability distribution or will gain an area lower than 10% of their current climatic suitability distribution. The balance (difference between gain and loss) in the climatic suitability distribution was negative and statistically different from zero in most scenarios (Table 1, Fig. 1), indicating more potential future loss than gain in the climatic suitability distribution area. The balance in the climatic suitability distribution area differed among *Shared Socioeconomic Pathways* (ANOVA using species as blocks for 2060 models: F = 6.88, p < 0.01; and 2100 models: F = 30.19, p < 0.01, Fig. 1), indicating the strength of the reduction in the climatic suitability distribution is related to the severity of climate change. The most significant reductions are expected in the scenarios SSP2-4.5, SSP3-7.0, and SSP5-8.5 compared to SSP1-2.6 in 2060 and, with higher differences, in 2100 (Fig. 1, see Supplementary Table S1). Despite the general pattern of climatic suitability distribution reduction predicted in the future, a few individual species are projected to gain climatic suitability distribution area (see Supplementary Table S1).

**Table 1:** t-tests to evaluate if the balance in the distribution area was different from zero. Balance (%) is the difference between area gain and area loss predicted and is proportional to the current distribution area for Atlantic Forest endemic primate species (n = 19). Negative values indicate reduction in distribution size predicted for the future. Projections for all Shared Socioeconomic Pathways (SSP1-2.6, the most optimistic one, SSP2-4.5, SSP3-7.0 and SSP5-8.5, the most pessimist one) and two times in the future (2060 and 2100) without considering potential dispersal are presented.

		Balance (%)		t	p	n
		Mean	SD			
2060	SSP1-2.6	− 21.74	30.28	− 3.13	0.01	19
	SSP2-4.5	− 30.11	32.39	− 4.05	< 0.01	19
	SSP3-7.0	− 30.37	31.17	− 4.25	< 0.01	19
	SSP5-8.5	− 34.58	32.58	− 4.63	< 0.01	19
2100	SSP1-2.6	− 20.74	33.88	− 2.67	0.02	19
	SSP2-4.5	− 34.89	39.13	− 3.89	< 0.01	19
	SSP3-7.0	− 49.21	42.88	− 5.00	< 0.01	19
	SSP5-8.5	− 56.95	43.81	− 5.67	< 0.01	19

**Figure 2:**
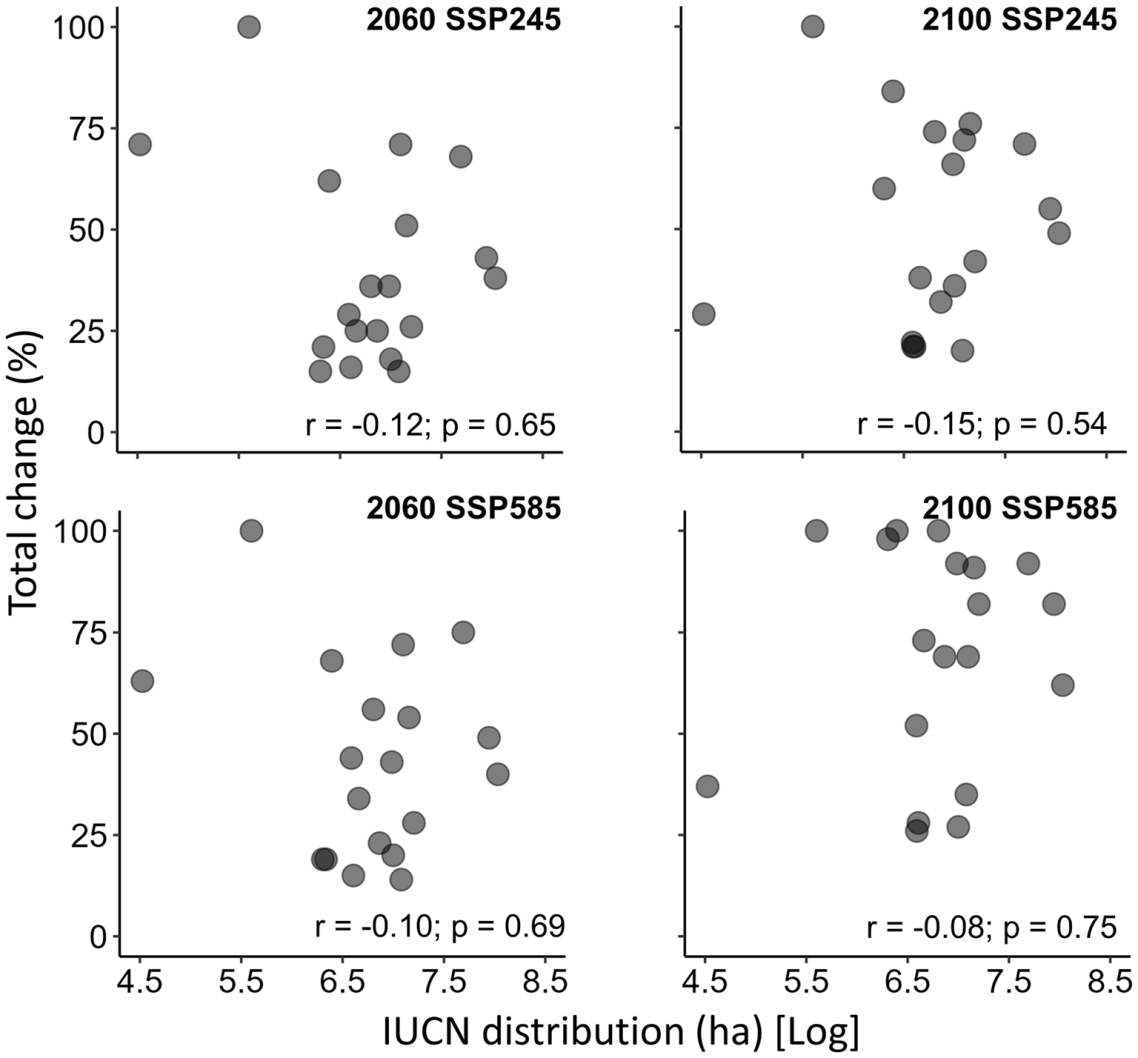
Scatterplots between the species’ IUCN geographical range and the relative total change expected in the climatic suitability area for Atlantic Forest endemic primate species (n = 19) in two *Shared Socioeconomic Pathways* (SSP2-4.5 and SSP5-8.5, the most pessimist one) for two times in the future (2060 and 2100) without considering potential dispersal. Total change is the sum of predicted area gain and area loss and is proportional to the current climatic suitability distribution area. Pearson correlation test results (*Pearson* r and p values) are show inside the graphs. We also run non-parametric Spearman correlation tests, with the same qualitative results (no correlation).

The species’ IUCN geographical range was not correlated with the relative total change expected in projected climatic suitability binarized maps (Fig. 2). This indicated that species with smaller geographical ranges, subject to less varied environmental conditions, are not predicted to have larger changes in their projected climate binarized maps from the current to the future.

**Figure 3:**
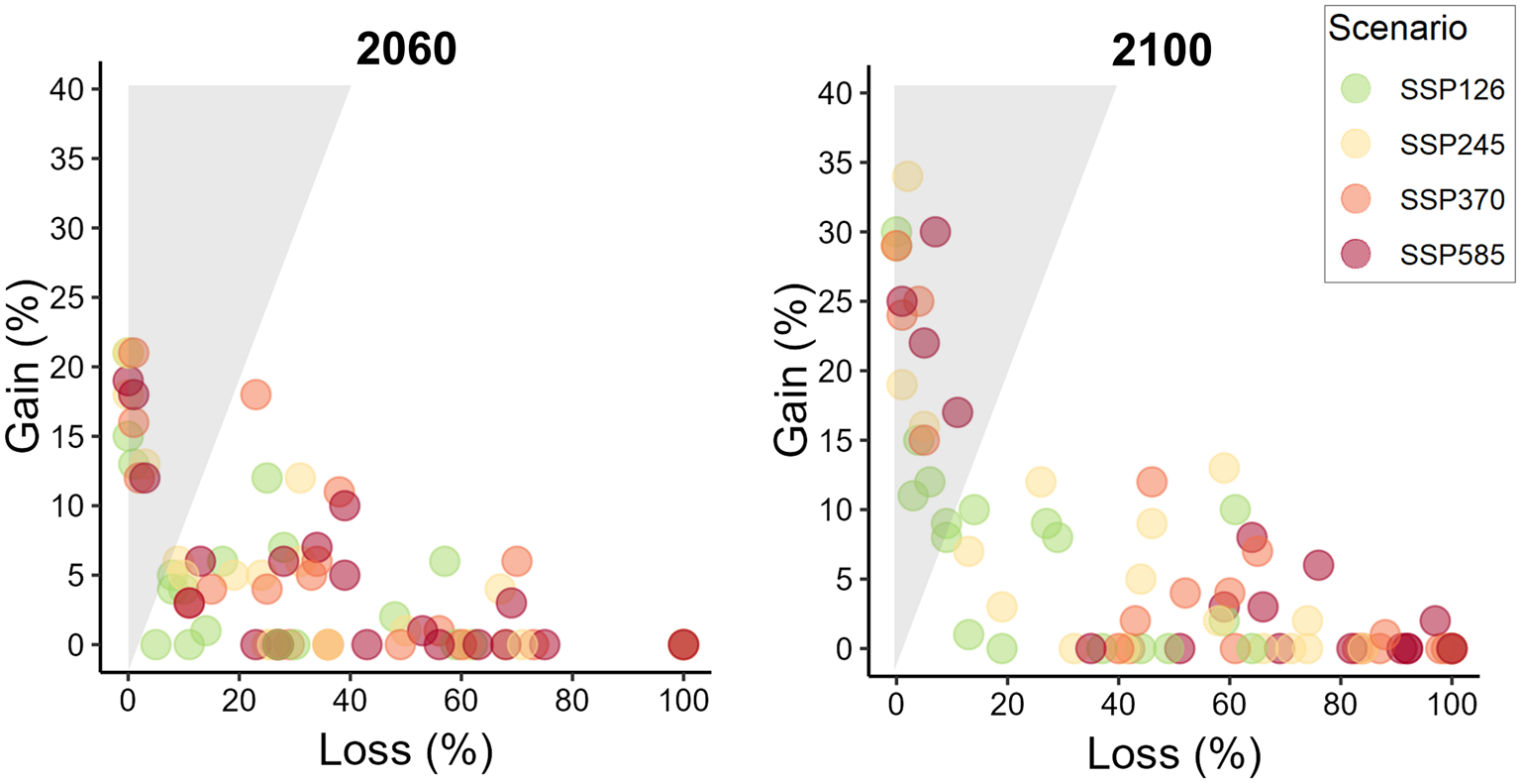
Relative climatic suitability area gain (%) and relative area loss (%) predicted for Atlantic Forest endemic primate species (n = 19) in all *Shared Socioeconomic Pathways* (SSP1-2.6, the most optimistic one, SSP2-4.5, SSP3-7.0 and SSP5-8.5, the most pessimist one) and for two times in the future (2060 and 2100) without considering potential dispersal. The shaded region represents more gain than loss proportions.

The median relative climatic suitability distribution loss among the species is four to 12 times higher (SSP 1–2.6 and SSP 5–8.5, respectively) than the median relative climatic suitability distribution gain in 2060 and two to 141 in 2100 (Table 2). A climatic suitability distribution loss is predicted for most Atlantic Forest primates (Fig. 3, see Supplementary Table S1), indicating they will lose much more than gain any area in the future. Nevertheless, the gain is predicted to be higher than the loss in the climatic suitability distribution for a few species (Fig. 3, points in the shaded region of the graph). Although detailed and profound analysis is needed for species-specific inferences, some results found in our community and general approach are as follows (Supplementary Fig. 1). We noticed the relative climatic suitability distribution loss predicted for 2060 is higher than 50%, while the gain is lower than 10% for *Callicebus nigrifrons*, *C. personatus*, *Callithrix flaviceps*, *C. geoffroyi*, *Leontopithecus caissara*, and *L. chrysopygus*. For the 2100 predictions, *Alouatta guariba*, *Brachyteles arachnoides*, *C. coimbrai, C. geoffroyi*, and *C. kuhlii* are predicted to lose between 50 and 74% of their current climatic suitability distribution, and *B. hypoxanthus*, *C. aurita*, *C. nigrifrons*, *C. personatus*, *L. chrysomelas,* and *S. nigritus* are predicted to lose more 75%. *Callithrix flaviceps* and *L. chrysopygus* do not have suitable areas in 2100 SSP 5–8.5 scenario, and *L. rosalia* does not have suitable areas in all 2060 and 2100 future scenarios. The species with higher relative climatic suitability distribution gain in 2060 and 2100 are *C. melanochir* (15–34%) and *S. flavius* (11–25%), and *A. belzebul* (15–17%), *L. caissara* (29–30%) in 2100 (see Supplementary Figure S1).

**Table 2:** Relative distribution area gain (%) and relative distribution area loss (%) predicted for endemic Atlantic Forest primates. Median and quartiles are presented for all *Shared Socioeconomic Pathways* (SSP1-2.6, the most optimistic one, SSP2-4.5, SSP3-7.0 and SSP5-8.5, the most pessimist one) for two times in the future (2060 and 2100) without considering potential dispersal.

		Relative area gain (%)			Relative area loss (%)		
		Q1	Median	Q3	Q1	Median	Q3
2060	SSP1-2.6	0.06	4.13	6.84	7.73	16.81	39.30
	SSP2-4.5	0.05	3.94	6.23	14.74	30.90	55.88
	SSP3-7.0	0.02	3.62	8.53	19.29	32.78	52.54
	SSP5-8.5	0.06	3.08	6.32	17.96	38.96	59.47
2100	SSP1-2.6	0.22	7.63	10.48	7.36	19.33	46.28
	SSP2-4.5	0.11	3.18	12.58	16.03	43.61	68.49
	SSP3-7.0	0.05	1.45	9.49	40.66	60.12	85.70
	SSP5-8.5	0.05	0.49	6.82	42.99	69.21	91.89

### Distribution shifts

**Figure 4:**
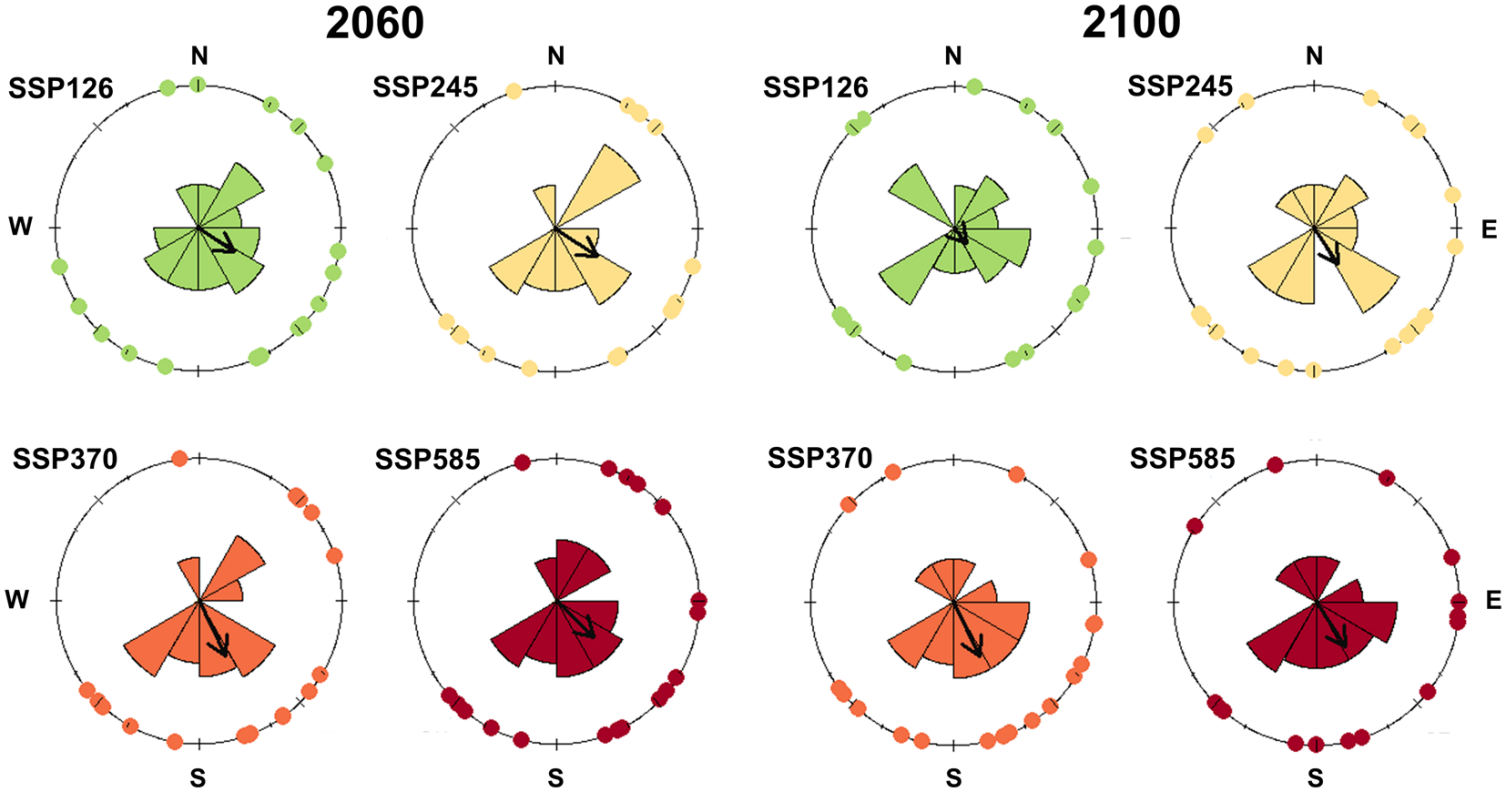
Circular plots showing the direction of climatic suitability distribution shifts for Atlantic Forest primate species (see Table 3 for information on the number of species) in all *Shared Socioeconomic Pathways* (SSP1-2.6, the most optimistic one, SSP2-4.5, SSP3-7.0 and SSP5-8.5, the most pessimist one) without considering potential dispersal. The circular mean is represented by the arrow. *Leontopithecus rosalia* does not have suitable areas in all 2060 and 2100 future projected scenarios and was not included in distribution shifts analysis (n = 18 in most ones). *Callithrix flaviceps* and *L. chrysopygus* do not have suitable areas in 2100 SSP 5–8.5 future projected scenario (n = 16). *Leontopithecus caissara* has the same centroid in suitable areas in current and 2060 SSP 1–2.6 future projected scenario (n = 17).

There were global directional shifts in climate distributions for Atlantic Forest primates only in SSP3-7.0 scenario in 2060 and 2100. Directional latitudinal shifts were detected specifically to the south in several scenarios (Table 3, Fig. 4). The mean centroid latitudinal shift projected is − 0.29 degrees (~ 32 km) for 2060 SSP3-7.0, − 0.44 degrees (~ 48 km) for 2100 SSP3-7.0, and − 0.46 degrees (~ 51 km) for 2100 SSP5-8.5 scenario.

**Table 3:** Results from the Rayleigh circular tests to evaluate global directional changes or specifically testing the alternative hypothesis or north, east, south, and west directional potential shifts for Atlantic Forest endemic primate distributions. Projections for all Shared Socioeconomic Pathways (SSP1-2.6, the most optimistic one, SSP2-4.5, SSP3-7.0 and SSP5-8.5, the most pessimist one) and two times in the future (2060 and 2100) without considering potential dispersal are presented. Once *L. rosalia* does not have suitable areas in all 2060 and 2100 future scenarios, it was not included in this analysis (no centroid information). N = 18 in most scenarios, exceptions 2060 SSP1-2.6 (N = 17, *L. caissara* excluded because the centroid was the same as current), and 2100 SSP5-8.5 (N = 16, *C. flaviceps* and *L. chrysopygus* does not have suitable areas in this scenario).

		Global test		H1: North		H1: East		H1: South		H1: West	
		Stat	*p*	Stat	*p*	Stat	*p*	Stat	*p*	Stat	*p*
2060	SSP1-2.6	0.30	0.20	− 0.16	0.83	0.25	0.07	0.16	0.17	− 0.25	0.93
	SSP2-4.5	0.35	0.11	− 0.19	0.87	0.29	0.04	0.19	0.13	− 0.29	0.96
	SSP3-7.0	0.43	0.04	− 0.38	0.99	0.19	0.13	0.38	0.01	− 0.19	0.87
	SSP5-8.5	0.36	0.09	− 0.25	0.93	0.26	0.06	0.25	0.07	− 0.26	0.94
2100	SSP1-2.6	0.13	0.75	− 0.10	0.72	− 0.08	0.32	0.10	0.28	− 0.08	0.68
	SSP2-4.5	0.30	0.19	− 0.25	0.93	0.17	0.15	0.25	0.07	− 0.17	0.85
	SSP3-7.0	0.42	0.04	− 0.38	0.99	0.19	0.13	0.38	0.01	− 0.19	0.87
	SSP5-8.5	0.37	0.11	− 0.31	0.96	0.20	0.13	0.31	0.04	− 0.20	0.87

All the results presented above are from no-dispersal scenarios. We chose to present these no-dispersal results in detail in the main text due to two main criteria. The first is the great importance of geographical barriers as limits to primate species distributions. Some examples are *Sapajus flavius* and *Alouatta belzebul*, both limited in the south by the São Francisco River, *Callicebus coimbrai*, limited in the north by São Francisco River, *C. nigrifrons*, limited in the west by Paraná and Paranaíba Rivers, and *S. robustus*, limited in the north by the Jequitinhonha River and in the south by Doce River. Moreover, the second, the significant habitat loss in the Atlantic Forest region (~ 29% of forest cover, heterogeneously distributed throughout the biome, https://mapbiomas.org/en?cama_set_language=en), which is also related to low connectivity in this biome, especially for species largely forest dependent. Below we will briefly describe the results considering the possibility of dispersal.

### Results considering potential dispersal possibility

Considering the possibility of dispersal, the balance (difference between gain and loss) in the climatic suitability distribution area was negative and statistically different from zero in the more emission scenarios SSP3-7.0 and SSP5-8.5 for 2100 (Supplementary Table S3, Supplementary Figure S2), indicating more potential future loss than gain in the distribution area (Supplementary Figure S3, Supplementary Table S4). In the other scenarios and all scenarios for 2060, the balance did not differ from zero. The balance differed among *Shared Socioeconomic Pathways* (ANOVA using species as blocks for 2060 models: F = 18.19, p < 0.01; and 2100 models: F = 23.15, p < 0.01, Supplementary Figure S2, Supplementary Table S1), indicating the strength of the reduction in the climatic suitability distribution is related to the severity of climate change. The species’ IUCN geographical range was negatively correlated with the relative total change expected in projected climatic suitability binarized maps, mainly in 2060 (Figure S4). This means species with smaller geographical ranges, subject to less varied environmental conditions, are predicted to have larger changes in their projected climatic suitability binarized maps from current to 2060 future. There were no directional shifts in climatic suitability distributions for Atlantic Forest primates (all Rayleigh circular tests not significative, Supplementary Table S5, Supplementary Figure S5). These potential dispersal results may be seen with caution once several primate species are limited by geographical barriers in some part of their distributions.

## Discussion

The climatic suitability distributions of Atlantic Forest primates are predicted to change in size and shift spatially due to climate changes. According to Graham et al.^43^, the Atlantic Forest will face climate warming, mainly increasing by more than 1 °C in temperature and decreasing by more than 4% in precipitation (for each degree plus), imposing those conditions on native primate species. Our projections confirmed the hypothesis that climatic suitability distribution areas would change. A forecast of the effect of climate change on the Atlantic Forest itself may enhance this threat^47^ projecting a reduction and shift to the south in the distribution of typical plant species, which may restrict primate feeding. Homogenization of woody plants^48^ may also restrict primate feeding. At the same time, the impact on protected areas^49^ can deteriorate existing protected populations.

We expected a change in climatic suitability distributions but without a clear hypothesis regarding the increase or decrease in climatic suitability distribution. The major latitudinal disposal of the Atlantic Forest may largely influence this perspective of no clear hypothesis. By presenting a wide variety of climatic conditions throughout its distribution, differences among regions can nullify the general analysis. Although, the balance in climatic suitability distribution area results for Atlantic Forest primates is negative, indicating more area loss than gain in the future. This distribution retraction will be more significant in the most pessimist climate change scenario (SSP5-8.5, both to 2060 and 2100). A large proportion of Atlantic Forest mammals will be unable to keep pace with climate change^9^.

Our results for Atlantic Forest primates include only climate change, but losses can even be greater if forest cover and forest cover changes are incorporated. The distributions of *Callicebus* species, mostly located in Atlantic Forest, will be drastically reduced by climate change, especially incorporating forest cover and potential habitat loss^20^. *Leontopithecus chrysomelas*, *L. chrysopygus*, and *L. rosalia* are also predicted to lose distribution area^44^. Response to climate change can be even worse than we predicted for *L. chrysopygus*, a primate species already facing a major reduction in habitat suitability^50^.

For Amazonian primates, most species distributions will increase if only the climate is considered, half of them will decrease if rivers are considered as dispersal barriers, and most of them will decrease if climate, barriers and habitat loss are included^36^. A recent study with 12 primate species endemic to the Amazon also addresses that condition, pointing out that four species will lose 90% of their range, increasing to eight species as deforestation is added into the analysis, with about three species losing more than 98% of their distribution^51^. The biotic velocity necessary to follow preferred climate conditions is larger than Amazonian primates’ maximum dispersal ability^52^. Only four species in our study will have more climatic suitability distribution gain than loss (although small). While a previous evaluation found range reduction for two of these species^53^, the results cannot be directly compared once the disjunct distribution of *Alouatta belzebul* in the Amazon was not considered in our study, and we limited the climatic suitability model for the current extent of occurrence (no dispersal scenario) or to a smaller dispersal distance.

The change in climatic suitability distribution predicted for the future is not related to the extent of occurrence. Once large distributions probably encompass higher spatial environmental variability, we expected a lower influence of temporal variability (current to future) on these distributions than smaller ones, but this was not corroborated. This relationship is important to species dealing with climate change once environmental and geographical variation can be surrogates for adaptive genetic variation and influences the adaptive potential^54,55^ and species’ capacity to tolerate threat changes across niche space^56^. L﻿ife history, genetic diversity, and external factors like the magnitude of climate change may be related to species’ capacity to track and respond to climate conditions changes^7^. Dispersal is also limited by landscape elements, such as patch connectivity^57^.

In our results, some species only lose climatic suitability distribution, and most lose more than they gain. Climate change’s loss in climatic suitability distribution is imminent for all species, and shifts in the climatic suitability distributions will characterize gains. Considering the 2060 predictions, the complete loss of climate suitability is predicted for one species (*Leontopithecus rosalia*), losing 100% of its current climatic suitability distribution without any gain. The complete loss is predicted for two more species for 2100 in SSP 5–8.5 scenario, *Leontopithecus chrysopygus*, and *Callithrix flaviceps*. A few exceptions with positive balances for SSP5-8.5 predictions are *Alouatta belzebul* (2060: gain 9% and loss 3%, 2100: gain 17% and loss 11%), *Callicebus melanochir* (2060: gain 19% and no loss, 2100: gain 22% and loss 5%), *Sapajus flavius* (2060: gain 12% and loss 6%, 2100: gain 38% and loss 1%), and *Leontopithecus caissara* (negative balance in 2060, 2100: gain 30% and loss 7%).

The area loss magnitude can compromise the species’ viability and increase threat status. For example, no area was predicted as suitable in the future above the threshold value for *L. rosalia*. This means that no area inside the species’ IUCN geographical range will have a future climate like those where it occurs nowadays, even considering potential dispersal. The distribution and suitability reduction were previously projected for *Leontopithecus* species^44,50^. Also, great proportional losses are more worrying in species that already encompass smaller geographic ranges, such as *Leontopithecus caissara* (current status as Critically Endangered, CR) and *L. rosalia* (Endangered, EN) (IUCN geographical range with less than 500,000 ha), *L. chrysomelas* (EN), *Callithrix flaviceps* (CR), *Callicebus coimbrai* (EN), *Sapajus flavius* (EN), *Alouatta belzebul* (Vulnerable, VU), *C. kuhlii* (VU), *L. chrysopygus* (EN), *Brachyteles arachnoides* (CR), *B. hypoxanthus* (CR) and *C. melanochir* (VU) (less than 10,000,000 ha). Some of these are predicted to lose more than 75% of their current distribution in 2100, considering the SSP5-8.5 scenario, such as *C. flaviceps*, *L. chrysopygus*, and *L. rosalia* (extinction predicted), *L. chrysomelas* (95% loss predicted, remaining ~ 87,000 ha), and *B. hypoxanthus* (92% loss predicted, remaining ~ 751,000 ha). Even species with larger extents are predicted to lose more than 75% of their current range, such as *C. nigrifrons* (92% loss predicted, remaining ~ 1,776,000 ha), *C. personatus* (91% loss predicted, remaining ~ 842,650 ha), and *C. aurita* (82% loss predicted, remaining ~ 2,139,000 ha). This turn lights the hard effects of climate change on some species and the need for further detailed and specific studies.

Our study with Atlantic Forest primates indicates climatic suitability distribution centroids moving southwards in some scenarios (2060: SSP1-2.6 and SSP3.7–0, 2100: SSP3-7.0 and SSP5-8.5). A meta-analysis showed shifts to higher latitudes due to climate changes at a median rate of 16.9 km decade^−1^^26^. There is still needs to be more studies about range shifts due to climate change in terrestrial environments of the Southern Hemisphere^58^. Potential dispersals to the south due to climate change were previously predicted for *Leontopithecus*^44^ and trees used by these primates^59^, as well as other tree species typical for Atlantic Forest^47^. The Neotropical primates depend on forested habitats for establishing populations. The possibility of dispersal climatically suitable areas in the future will depend on suitable routes^60^ and landscape configuration^61^. Beyond that, species dispersal capacity needs to be equivalent to or higher than the spatial velocity of climate change^62^.

Our evaluation projects only climate change effects on species climatic suitability distributions, indicating a general greater loss in more pessimistic scenarios. While the balance was negative (indicating more loss than gain in distribution) in all scenarios without potential dispersal, in scenarios with dispersal, this balance is negative only in 2100 SSP3-7.0 and SSP5-8.5. Unlike no dispersal scenarios that indicated southward distribution shifts, potential dispersal ones predicted no directional spatial change. In no dispersal scenarios, models were cropped by species’ IUCN geographical ranges, encompassing specialist knowledge about geographic barriers, such as rivers. The two approaches are informative once some species are limited by geographical barriers in some parts of their distributions, but not all of them.

The habitat suitability projection in the future assumes the maintenance of the model adjusted using current climate data. This is reasonable for large-bodied species for which the number of generations in the given time will be small, decreasing the chance for acclimation, even more for any possibility of evolution and adaptation. Besides, acclimation for marginal populations along distribution is complex, mostly for those on the opposite side of the shifting direction. Those populations may face extinction, resulting in distribution loss, especially in fast climate change conditions and habitat loss, narrowing gene flow^4^.

The reduction in species’ climatic suitability distribution will reflect their threatened status, mainly those already restricted to small distributions, as proposed by Davies et al.^63^. Conversely, species with broader distributions are commonly generalists with better dispersal capacity^64^. Although, their dispersal will be limited by habitat reduction and fragmentation, a current reality in the Atlantic Forest. More than half of the primate species here evaluated are Endangered or Critically Endangered. Our results emphasize that no more natural forest loss is acceptable, and restoration is urgent to deal with climate change.

The shifts and losses in climatic suitability distributions will lead species to much higher extinction risk in the worst climate change scenario. So, decreasing greenhouse gas emissions is essential to reduce the speed of climate change to facilitate and increase the chances of species adaptation or acclimatization. The speed of climate change is so high that it would require more significant rates of adaptive evolutionary change than historically registered for primates^65^. The primates' success in leading with climate changes depends on impending habitat loss in the Atlantic Forest. The maintenance and protection of highly genetically distinct populations^66^, restoration mainly of riparian areas, increase in forest patch connectivity within and across properties, as well as the building of canopy bridges in specific conditions^67^ may integrate the reactive approaches. According to Piffer et al.^68^, riparian restored forests are among the greater longevity areas of restoration persistence along the Atlantic Forest and forests in industrial agricultural areas. This last condition may result from obligatory maintenance of “legal reserves” (mandatory protected areas within properties), those with continuous surveillance. Poor remnants currently occupied or projected to be climatically suitable in the future could also be enriched with native plant species used by primates as resources. Additional measures to benefit population maintenance and dispersal are political and economic subsidies to more friendly agroecosystems^69^, monitoring infrastructure projects, and implementing measures to facilitate individual displacement (such as canopy bridges^70^). Species with a drastic reduction in their distributions may be supported with specific conservation decisions, such as forest restoration and animal translocation, called Reactive Approaches^71^. The same authors also indicate Proactive Approaches, such as establishing protected areas. An additional important step is including anthropogenic climate change as a threat factor, such as recently done for *Leontopithecus chrysopygus*^72^.

## Materials and methods

We evaluated the effects of climate change on 19 primate species (encompassing six genera) that have ~ 50% or more of their geographical range (according to IUCN, accessed in May/2021) in the Atlantic Forest. Two of the six genera are endemic to the Atlantic Forest (*Brachyteles*, two species, and *Leontopithecus*, four species). Among the 19 species, four are classified as Critically Endangered, seven are Endangered, two are Near Threatened, five are Vulnerable, and one is Least Concern (see Supplementary Table S6, IUCN, accessed in July/2021).

### Current and future habitat suitability

Occurrence records used to construct habitat suitability models for each species were compiled from published papers indexed in *Web of Science* and *Scielo* (using genus names as keywords in search, more details described in^46^) and published in the specialized journals *Neotropical Primates* and *Checklist* (all volumes and numbers, more details described in^46^) and from a data paper^73^. We filtered the point locality data from the data paper by removing inaccurate data, such as localities where the species is exotic, hybrids, or data not georeferenced.

We defined each species’ potential dispersal geographic limit, adding a maximum dispersal distance buffer to the species’ IUCN geographical range (Supplementary Figure S6). For each species, we developed the following procedure. First, the dispersal distance in one generation time was estimated according to Eq. (1)^85^,using home range data obtained from^86^:

1 $$Dispersal\; distance\; in\; one\; generation = 40 \times \sqrt {home\; range}$$

We obtained the generation time from^87^. Then, the number of generations from 1985 to 2050 (65 years) and from 1985 to 2090 (105 years) was calculated by dividing the time intervals by the generation time. Finally, the maximum dispersal distance in each time interval was obtained by multiplying the dispersal distance in one generation by the number of generations in the time interval (2).

2 $$Maximum\; dispersal\; distance = dispersal\; distance\; in\; onne\; generation \times number\; of\; generations$$

We defined a buffer of potential dispersal geographic limit beyond the IUCN geographical range using the maximum dispersal distance. For each species, we obtained two potential dispersal geographic limits, one for a 65 years time interval and one for a 105 years time interval (see Supplementary Figure S6 and Supplementary Table S7).

Current bioclimatic spatial data were used to adjust habitat suitability models. These variables were obtained from the WorldClim v2.1 database (^74^, https://www.worldclim.org/data) in a 2.5’ spatial resolution. These bioclimatic variables include several temperature and precipitation attributes (https://www.worldclim.org/data/bioclim.html). The potential dispersal geographic limits in 105 years of all the 19 species were summed to generate the geographical extent used in modeling. Bioclimatic variables were cropped to this extent, and background points were randomly sampled over this extent. Only those bioclimatic variables with variation inflation factor^75^ ≤ 3^76^ were kept to avoid multicollinearity*.* After this procedure, we maintained the bioclimatic variables BIO2 (Mean Diurnal Range of the Temperature), BIO8 (Mean Temperature of Wettest Quarter), BIO9 (Mean Temperature of Driest Quarter), BIO13 (Precipitation of Wettest Month), BIO18 (Precipitation of Warmest Quarter) and BIO19 (Precipitation of Coldest Quarter). We maintained only one occurrence data per pixel to reduce spatial bias. The habitat suitability models were adjusted using Maxent^77,78^. This algorithm showed good performance compared to 16 alternative algorithms^79^. The ‘*block*’ partition of training and testing data was used for species with more than 25 occurrence data, and the ‘*n-1 Jackknife*’ one for species with less than 25 occurrence data^80–83^. Occurrence data is equally partitioned in four bins based on its latitude and longitude distribution in ‘*block*’ method, and each of *n* occurrence points is used for testing once in the ‘*n-1 Jackknife*’^80^. Sixty models were adjusted for each species, combining ten *regularization multipliers* and six *feature classes*, and the model with the lower corrected Akaike Information Criterion (AICc) and the Area Under the ROC Curve (AUC) value higher than 0.75^80^ was selected (see Supplementary Table S8).

Habitat suitability was spatially projected for each species using current and future climate data. Future climate data were obtained from the same WorldClim v2.1 database (^74^ https://www.worldclim.org/data) in a 2.5’ spatial resolution. Habitat suitability in the future was projected considering each *Shared Socioeconomic Pathways* (SSP1-2.6, the most optimistic one, SSP2-4.5, SSP3-7.0 and SSP5-8.5, the most pessimist one) for 2060 (2041–2060) and 2100 (2081–2100), using eight Global Circulation Models (GCMs: BCC-CSM2-MR; CanESM5; CNRM-CM6-1; CNRM-ESM2-1; IPSL-CM6A-LR; MIROC6; MIROC-ES2L e MRI-ESM2-0). For each SSP and future time, we built a consensus model calculating the arithmetic average of the eight GCMs. Five final habitat suitability models (current and four future projections for each future time) were binarized using the *10th percentile training presence threshold* (see Supplementary Table S8).

We cropped the current climate binarized models for each species by the IUCN geographical range (IUCN, spatial data downloaded in 2020). Following that, for each species, we cropped the future climate binarized models according to 1) the IUCN geographical range in a scenario without dispersal, once this indirectly incorporates limitation factors that influence the distributions, such as geographical barriers and congeners interactions; 2) the potential dispersal geographic limit.

In the end, we have 17 climatic suitability models binarized: the current one and 16 future ones projected in two times x two dispersal scenarios x four shared socioeconomic pathways (SSPs).

### Measuring and analyzing distribution changes

**Figure 5:**
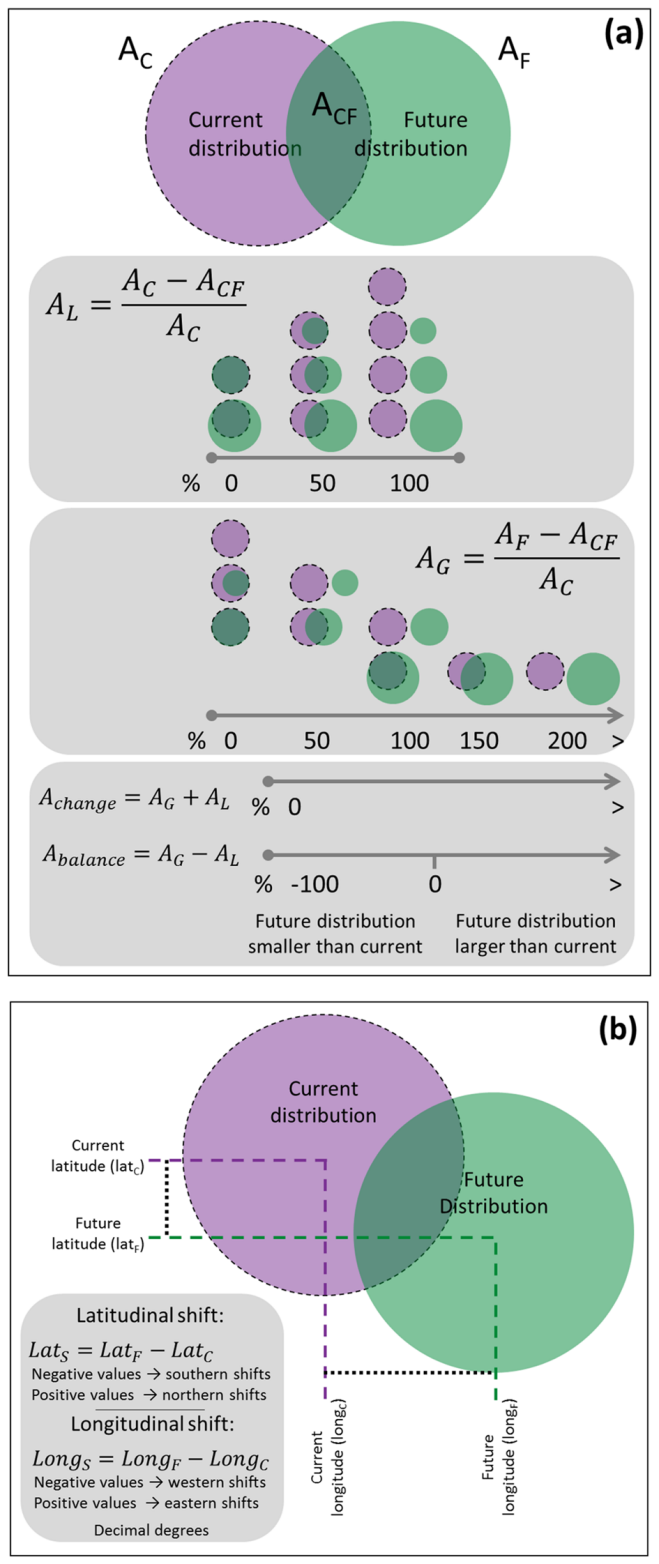
Measuring potential changes in area (**a**) and shifts (**b**) in species’ distributions due to climate change. Area of the current ($A_{C}$) and the future ($A_{F}$) distributions, area of the intersection between the current and future distributions (A_CF_), relative area loss in the future ($A_{L}$), relative area gain in the future ($A_{G}$), relative total change in the area ($A_{change}$), relative balance in gain and loss ($A_{balance}$), latitudinal shift ($lat_{S}$), and longitudinal shift ($long_{S}$).

To investigate if the species’ climate range will change (i.e., reducing, expanding, shifting), we measured potential changes in the area of climatic suitability models binarized (from now on called ‘distributions’) from the current time to the future. We measured the area of the current (A_C_, in ha) and the future (A_F_, in ha) distributions; the area of the intersection between the current and future distributions (A_CF_, in ha); the relative area loss in the future $\left( {A_{L} = \frac{{A_{C} - A_{CF} }}{{A_{C} }} \times 100} \right)$; the relative area gain in the future $\left( {A_{G} = \frac{{A_{F} - A_{CF} }}{{A_{C} }} \times 100} \right)$; the relative total change in the area ($A_{change} = A_{G} + A_{L}$); the relative balance in gain and loss ($A_{balance} = A_{G} - A_{L}$) (Fig. 5a). We tested whether the relative balance in the area was different from 0 (zero) using a t-test. Positive values indicate more area gain, and negative values indicate more area loss in distributions. We also evaluated if the severity of the climatic scenarios influences the potential changes in distribution testing if the balance were different among climate scenarios with an analysis of variance, using species as blocks.

To investigate if the potential changes in climatic distribution areas are related to the IUCN geographical ranges, we tested the correlation (*Pearson* and *Spearman*) between the geographical extent and the relative total change in the area.

To investigate the main trend in direction shifts of the species’ distributions, we measured the latitudinal shift by the difference between future and current latitude centroids ($lat_{S} = lat_{F} - lat_{C}$), and the longitudinal shift by the difference between future and current longitude centroids ($long_{S} = long_{F} - long_{C}$) (Fig. 5b). We evaluated the range shifts in direction using Rayleigh circular tests.

Habitat suitability modeling (‘ENMeval’ and ‘dismo’ packages) and statistical analysis (‘stats’ and ‘circular’ packages) were run in R software^88^. We used QGIS^89^ for processing and visualizing spatial datasets. We run the analysis for all the SSP scenarios projected for 2060 and 2100, considering no-dispersal and potential dispersal. We will present detailed results (including main figures and tables) related to the no dispersal scenarios in the main text. The results related to the potential dispersal scenario will be briefly described in the last paragraph of the results, and the detailed figures and tables can be accessed in the Supplementary Material.

## Supplementary Information

Supplementary Information 1.

Supplementary Information 2.

## Data Availability

The datasets generated during and/or analysed during the current study are available from the corresponding author upon reasonable request.
