# Supplementary material for: Primates facing climate crisis in a tropical forest hotspot will lose climatic suitable geographical range
Source: Sci Rep. 2023 Jan 12;13:641. doi: 10.1038/s41598-022-26756-0 (PMC9837198; doi:10.1038/s41598-022-26756-0)
Supplement: Supplementary file 1 — Supplementary Information 1. [file 41598_2022_26756_MOESM1_ESM.pdf]

Supplementary Material Figure S1

This Supplementary Material Figure S1 includes plates with maps of projected current and future climatic suitability maps (binarized).

We also pointed out in the plates the rivers that are potential barriers when it is the case. The information about the rivers was obtained from the IUCN geographic range description of each species (accessed in November/2022).

The maps were generated using QGIS 3.22.9-Bialowieza (https://www.qgis.org/en/site/)

Brazilian states abbreviations that appear in the plates are listed below.

AL - Alagoas
BA - Bahia
CE - Ceará
ES - Espírito Santo
GO - Goiás
MA - Maranhão
MG - Minas Gerais

MT - Mato Grosso
MS - Mato Grosso do Sul
PA - Pará
PB - Paraíba
PE - Pernambuco
PI - Piauí
PR - Paraná

RJ - Rio de Janeiro
RN - Rio Grande do Norte
RS - Rio Grande do Sul
SC - Santa Catarina
SE - Sergipe
SP - São Paulo
TO - Tocantins

In the maps we used the following shapefiles.
IUCN* species' geographical range (download from https://www.iucnredlist.org/ in May/2021)
IBGE** Brazilian states map (download https://ibge.gov.br/geociencias/organizacao-do-territorio/malhas-territoriais/15774-malhas.html?=&t=downloads in February/2022)
* The International Union for Conservation of Nature (IUCN)
** Instituto Brasileiro de Geografia e Estatística (IBGE)

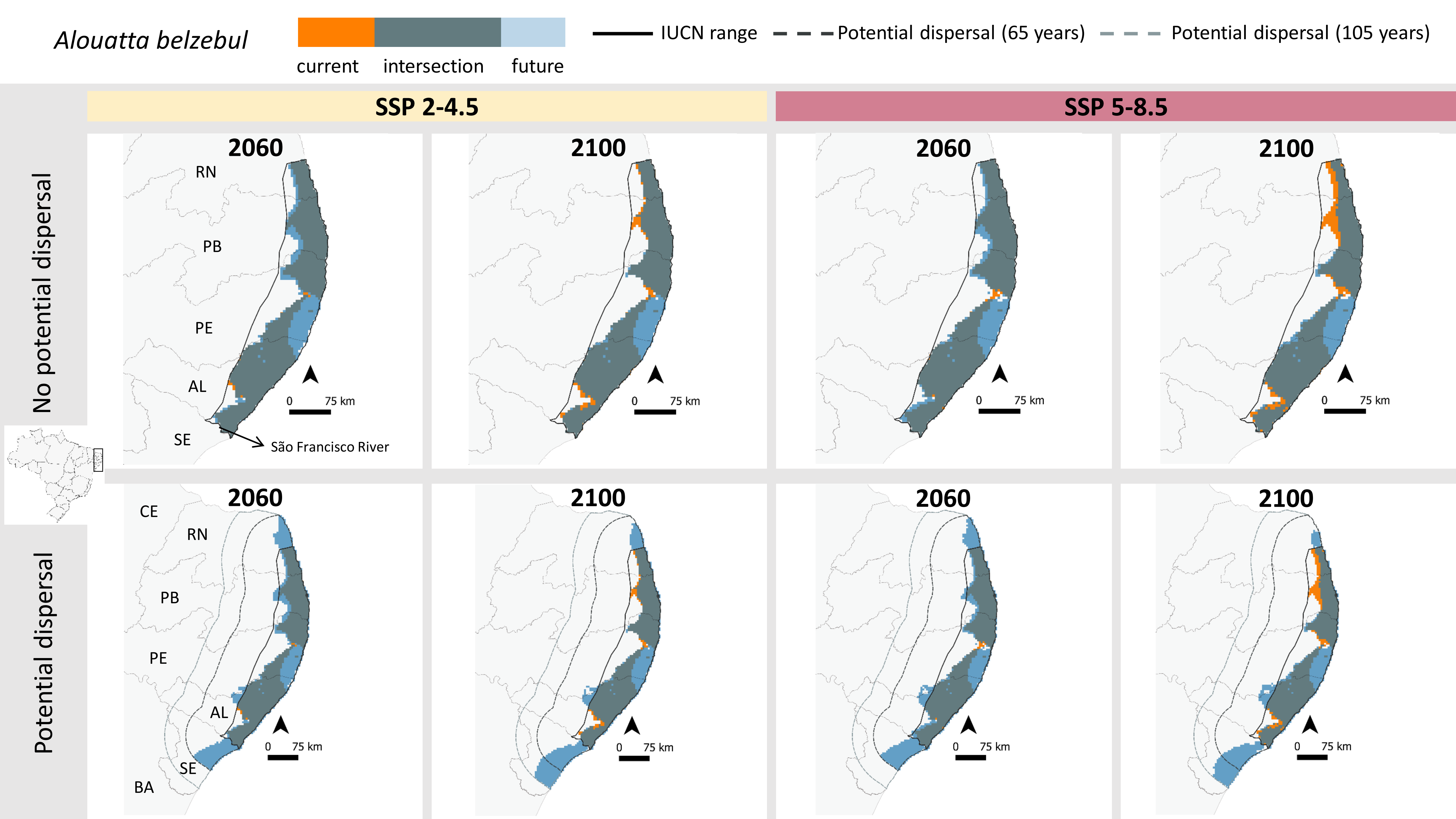

Alouatta belzebul
current
intersection
future
IUCN range
Potential dispersal (65 years)
Potential dispersal (105 years)
SSP 2-4.5
SSP 5-8.5
No potential dispersal
Potential dispersal
2060
2100
RN
PB
PE
AL
SE
CE
BA
São Francisco River
0
75 km

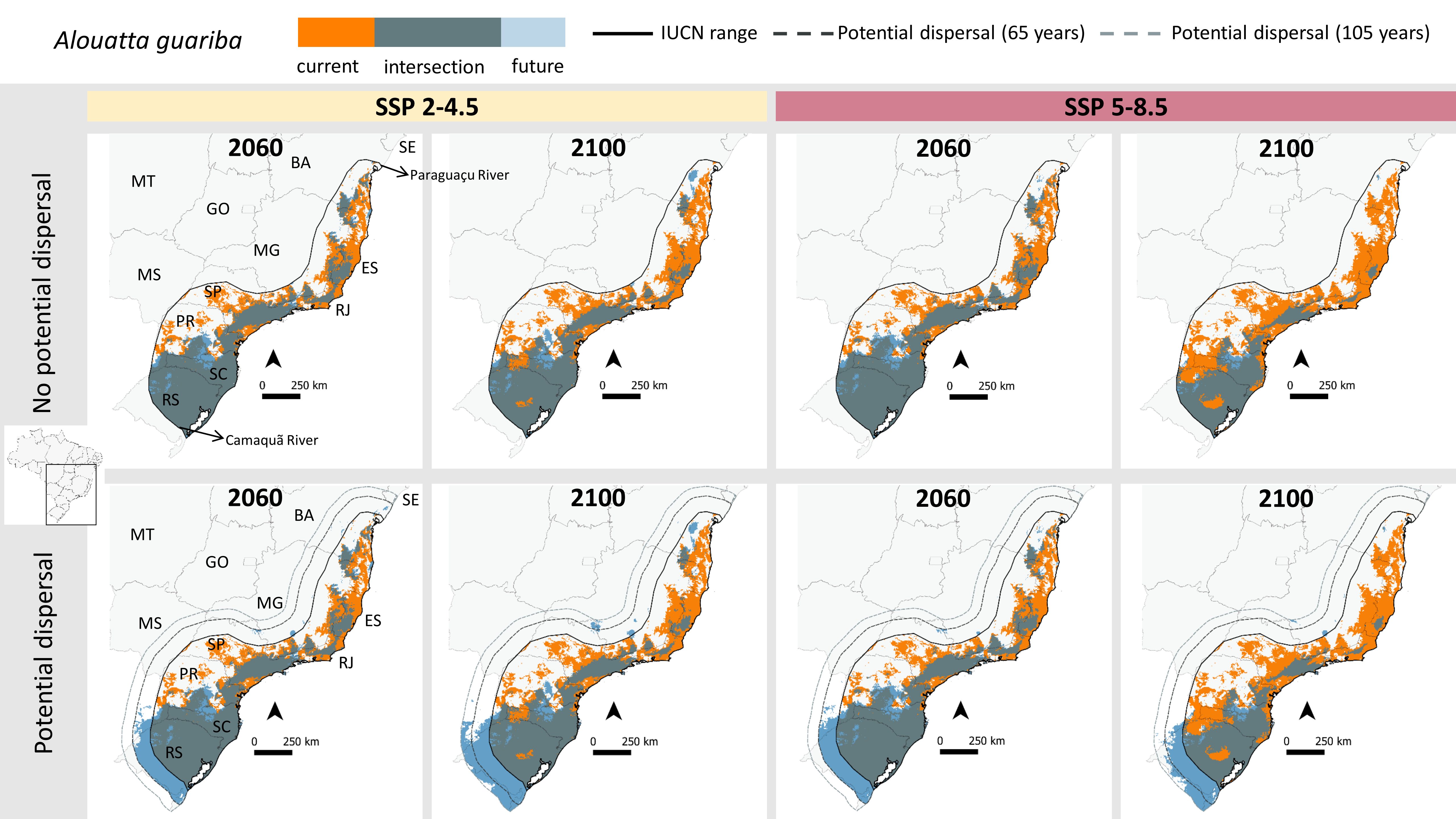

Alouatta guariba
current
intersection
future
IUCN range
Potential dispersal (65 years)
Potential dispersal (105 years)
SSP 2-4.5
SSP 5-8.5
No potential dispersal
Potential dispersal
2060
2100
2060
2100
MT
GO
MG
MS
BA
SE
ES
RJ
SP
PR
SC
RS
Paraguaçu River
Camaquã River
0 250 km

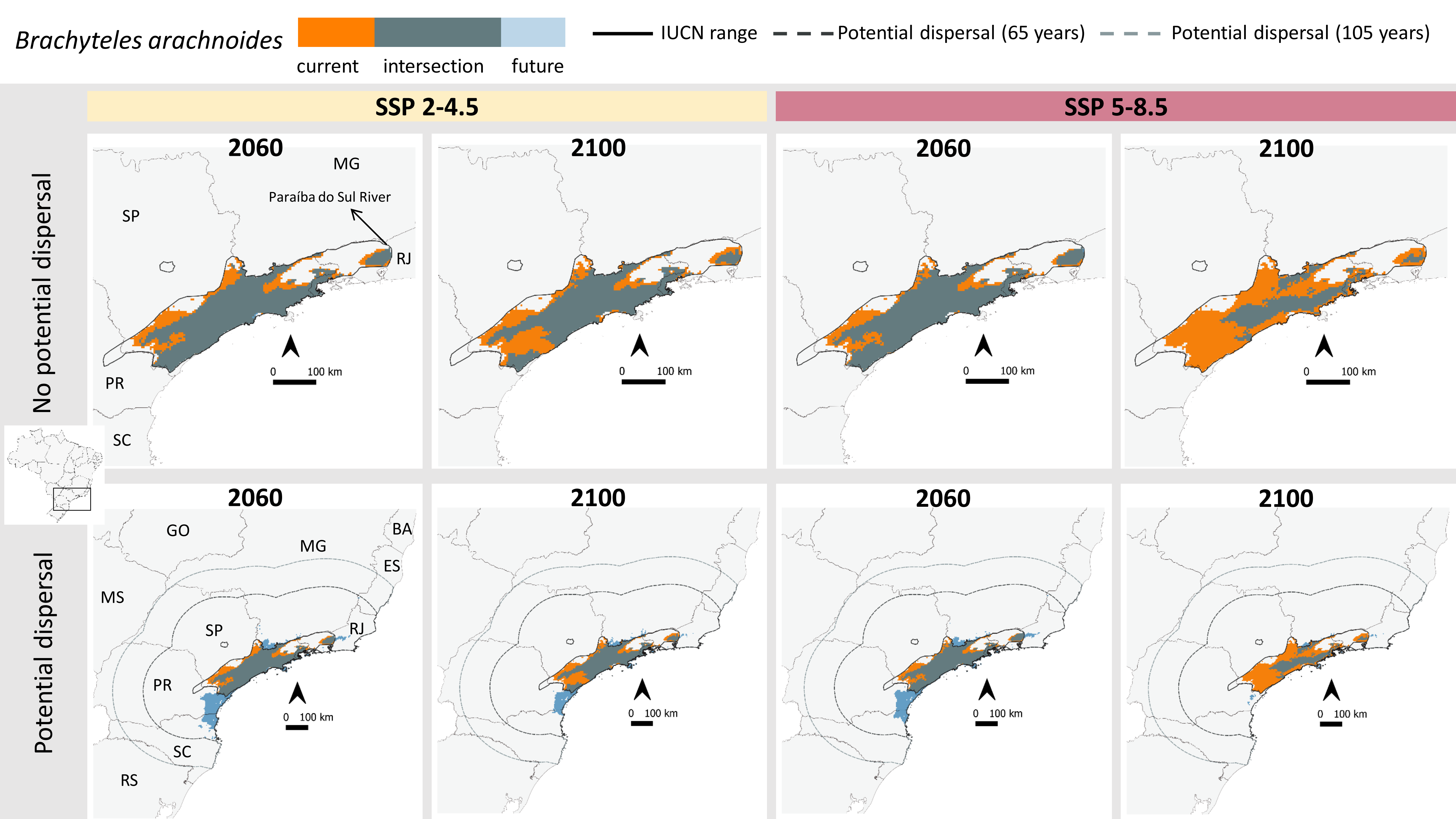
Brachyteles arachnoides
current
intersection
future
IUCN range
Potential dispersal (65 years)
Potential dispersal (105 years)
SSP 2-4.5
SSP 5-8.5
No potential dispersal
Potential dispersal
2060
2100
2060
2100
MG
SP
Paraíba do Sul River
RJ
PR
SC
0 100 km
2060
2100
2060
2100
GO
MG
BA
ES
MS
SP
RJ
PR
SC
RS
0 100 km

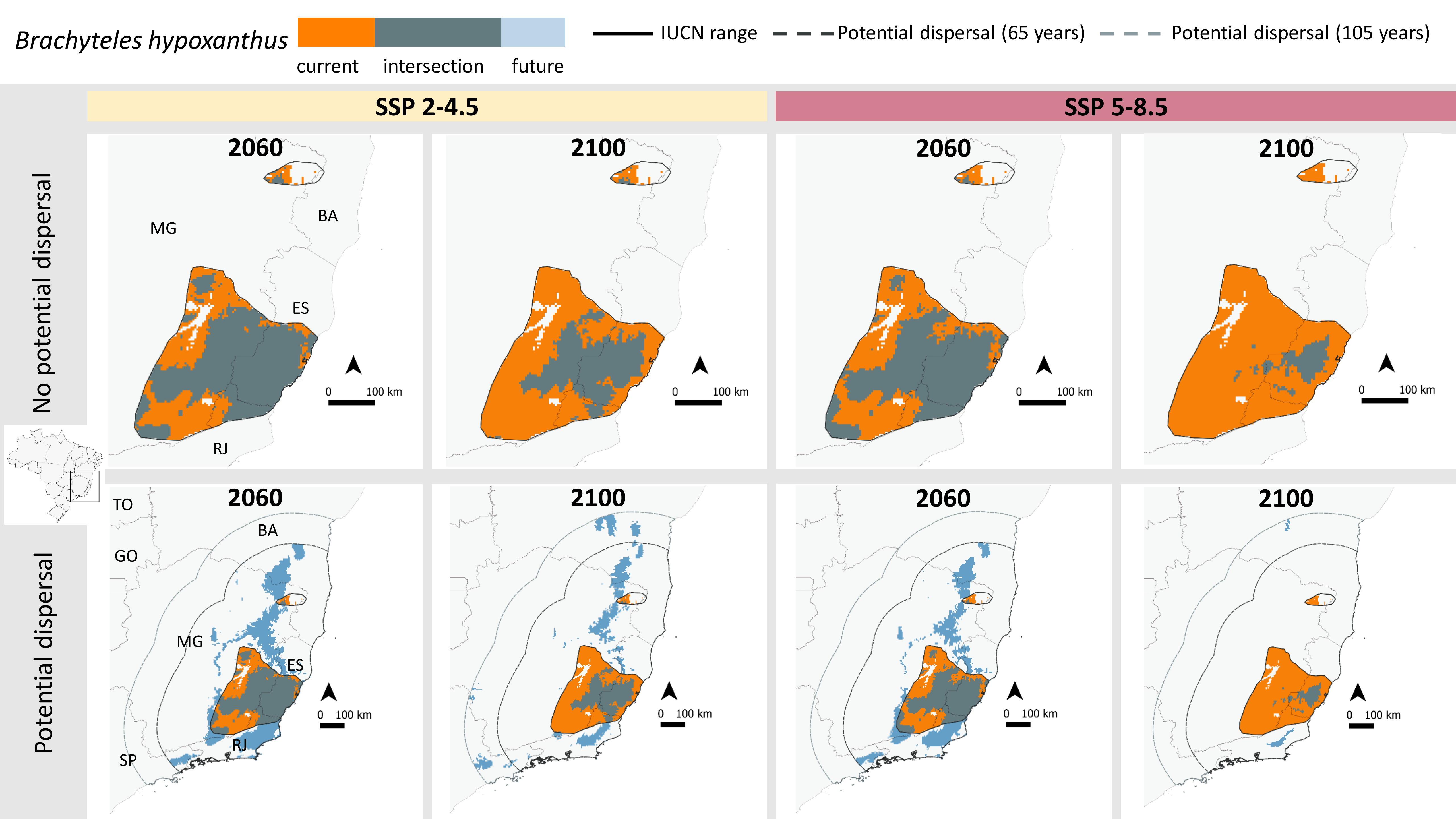
Brachyteles hypoxanthus
current
intersection
future
IUCN range
Potential dispersal (65 years)
Potential dispersal (105 years)
SSP 2-4.5
SSP 5-8.5
No potential dispersal
Potential dispersal
2060
2100
2060
2100
MG
BA
ES
RJ
0 100 km
2060
2100
2060
2100
TO
GO
BA
MG
ES
RJ
SP
0 100 km

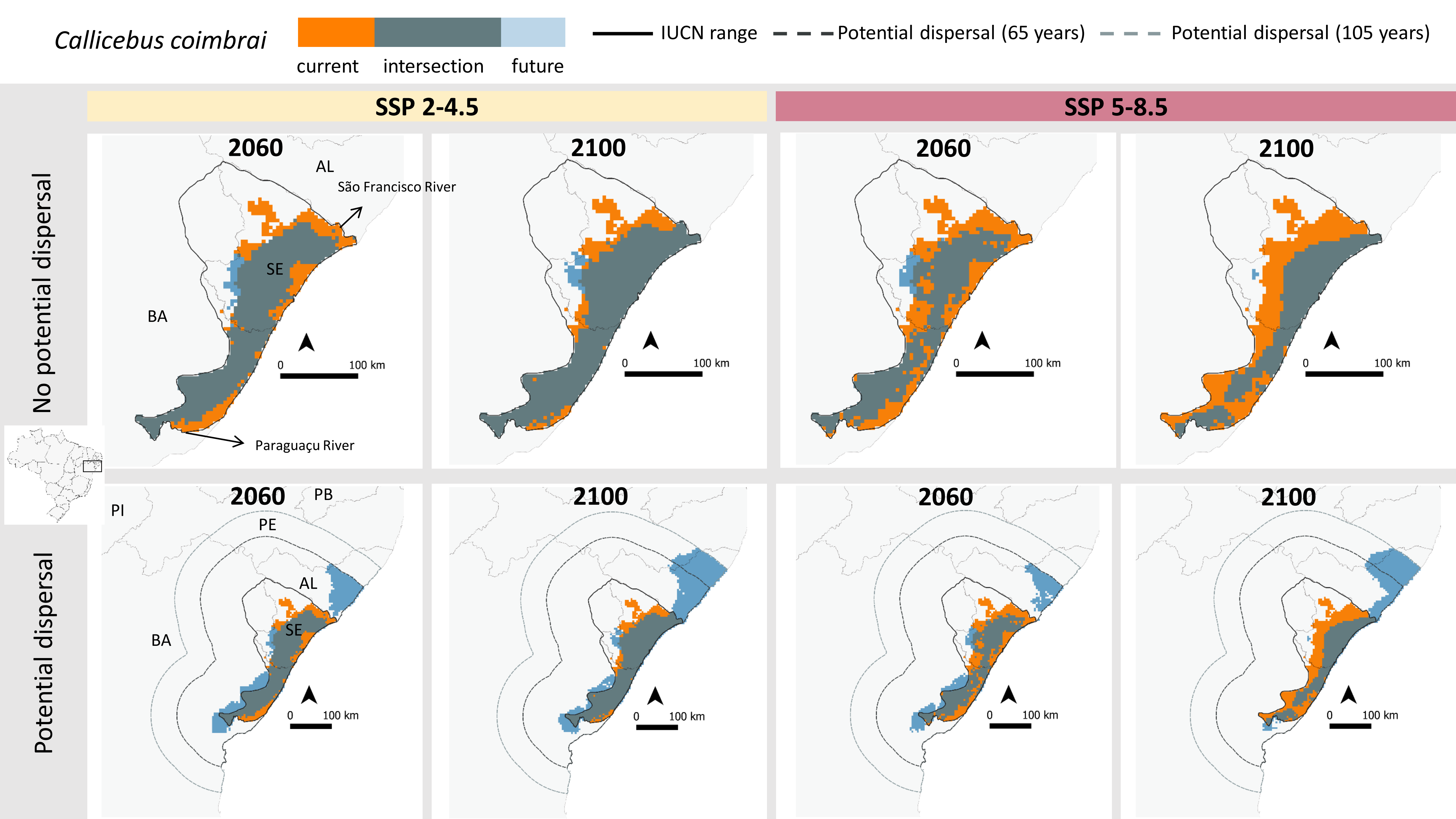

Callicebus coimbrai
current
intersection
future
IUCN range
Potential dispersal (65 years)
Potential dispersal (105 years)
SSP 2-4.5
SSP 5-8.5
No potential dispersal
Potential dispersal
2060
2100
AL
São Francisco River
SE
BA
Paraguaçu River
PB
PI
PE
0
100 km

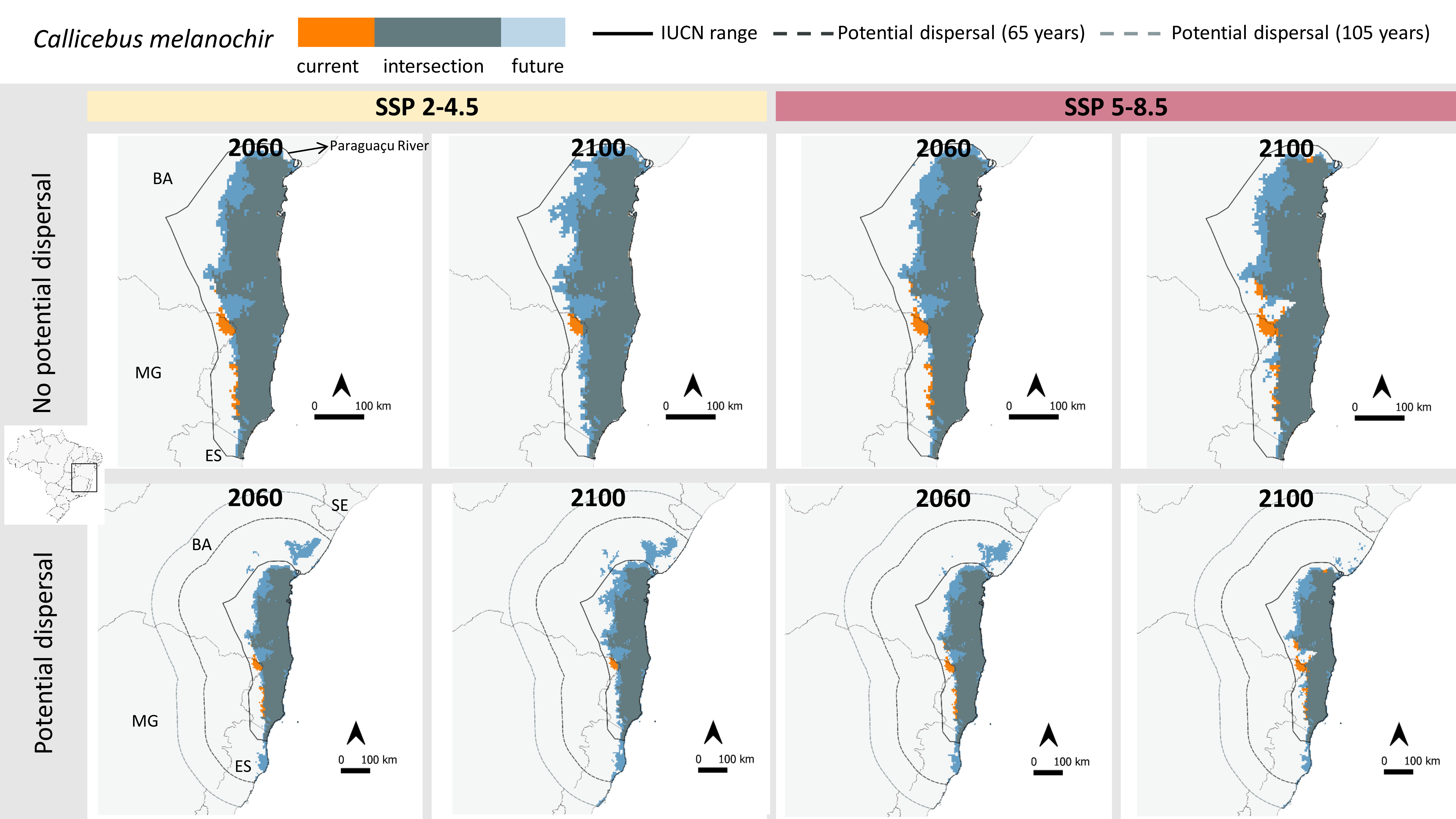

Callicebus melanochir
current
intersection
future
IUCN range
Potential dispersal (65 years)
Potential dispersal (105 years)
SSP 2-4.5
SSP 5-8.5
No potential dispersal
Potential dispersal
2060
2100
Paraguaçu River
BA
MG
ES
SE
0
100 km

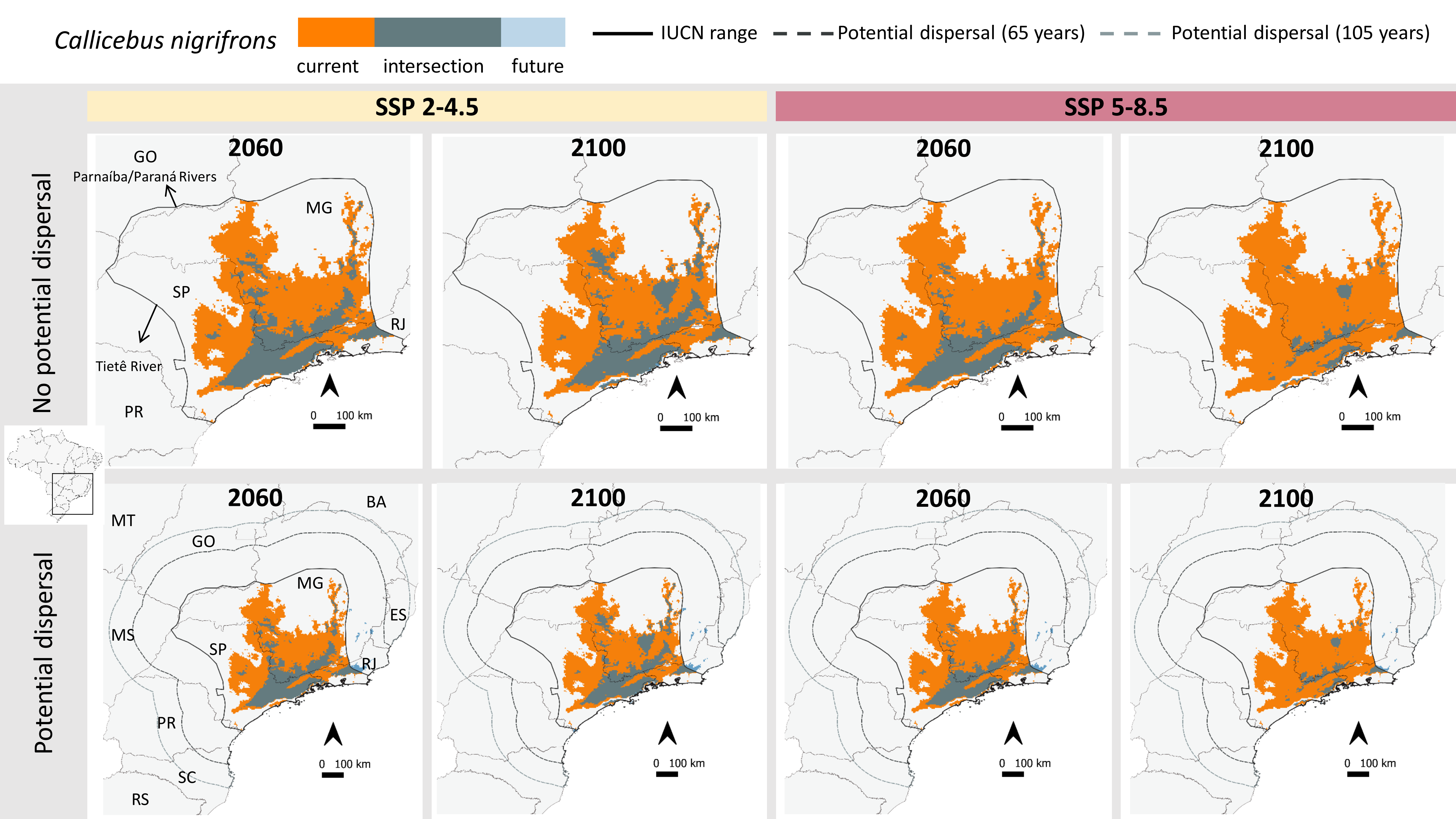

Callicebus nigrifrons
current
intersection
future
IUCN range
Potential dispersal (65 years)
Potential dispersal (105 years)
SSP 2-4.5
SSP 5-8.5
No potential dispersal
Potential dispersal
2060
2100
GO
Parnaíba/Paraná Rivers
MG
SP
RJ
Tietê River
PR
MT
BA
ES
MS
SC
RS
0 100 km

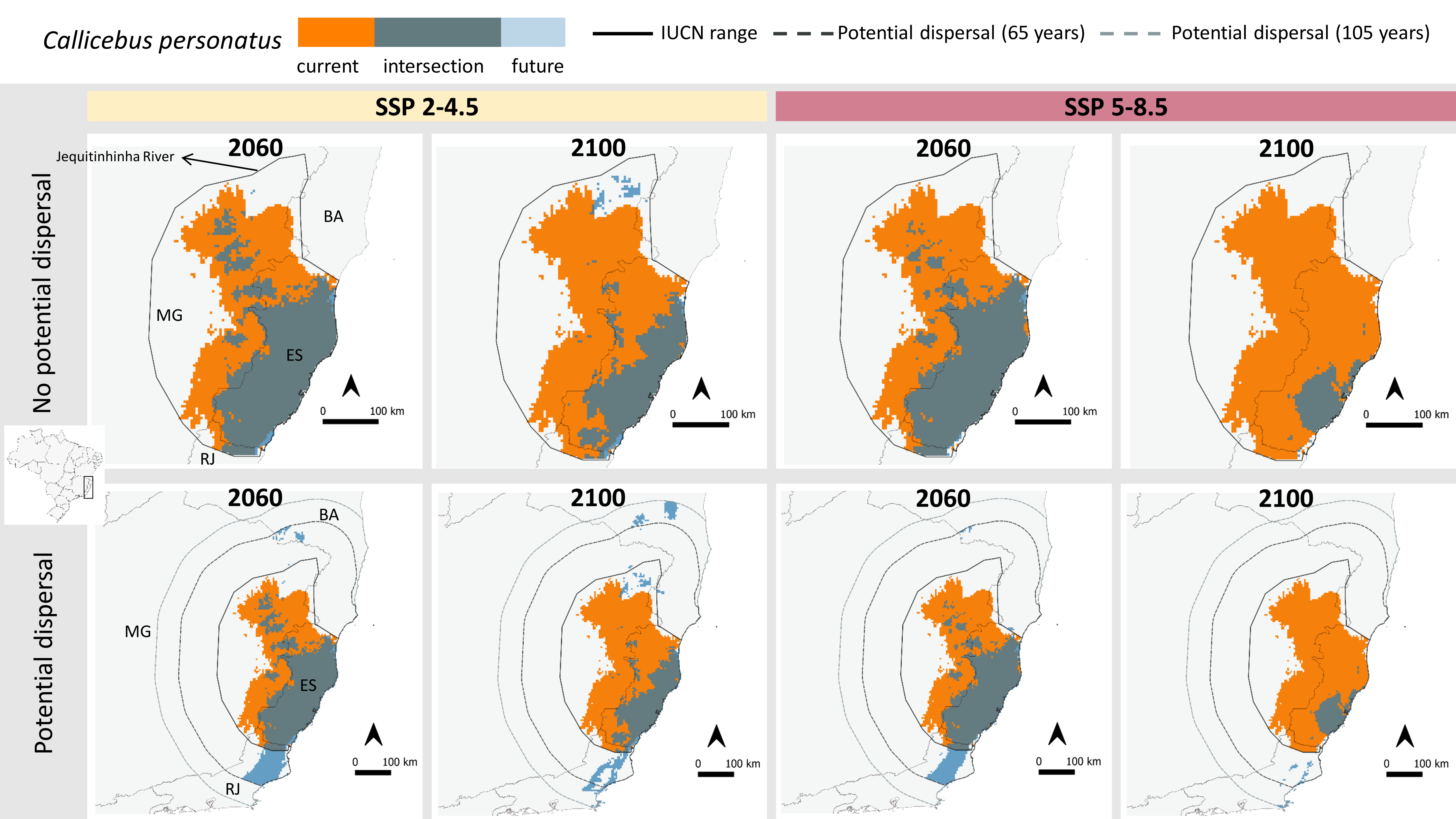

Callicebus personatus
current
intersection
future
IUCN range
Potential dispersal (65 years)
Potential dispersal (105 years)
SSP 2-4.5
SSP 5-8.5
No potential dispersal
Potential dispersal
2060
2100
Jequitinhinha River
BA
MG
ES
RJ
0
100 km

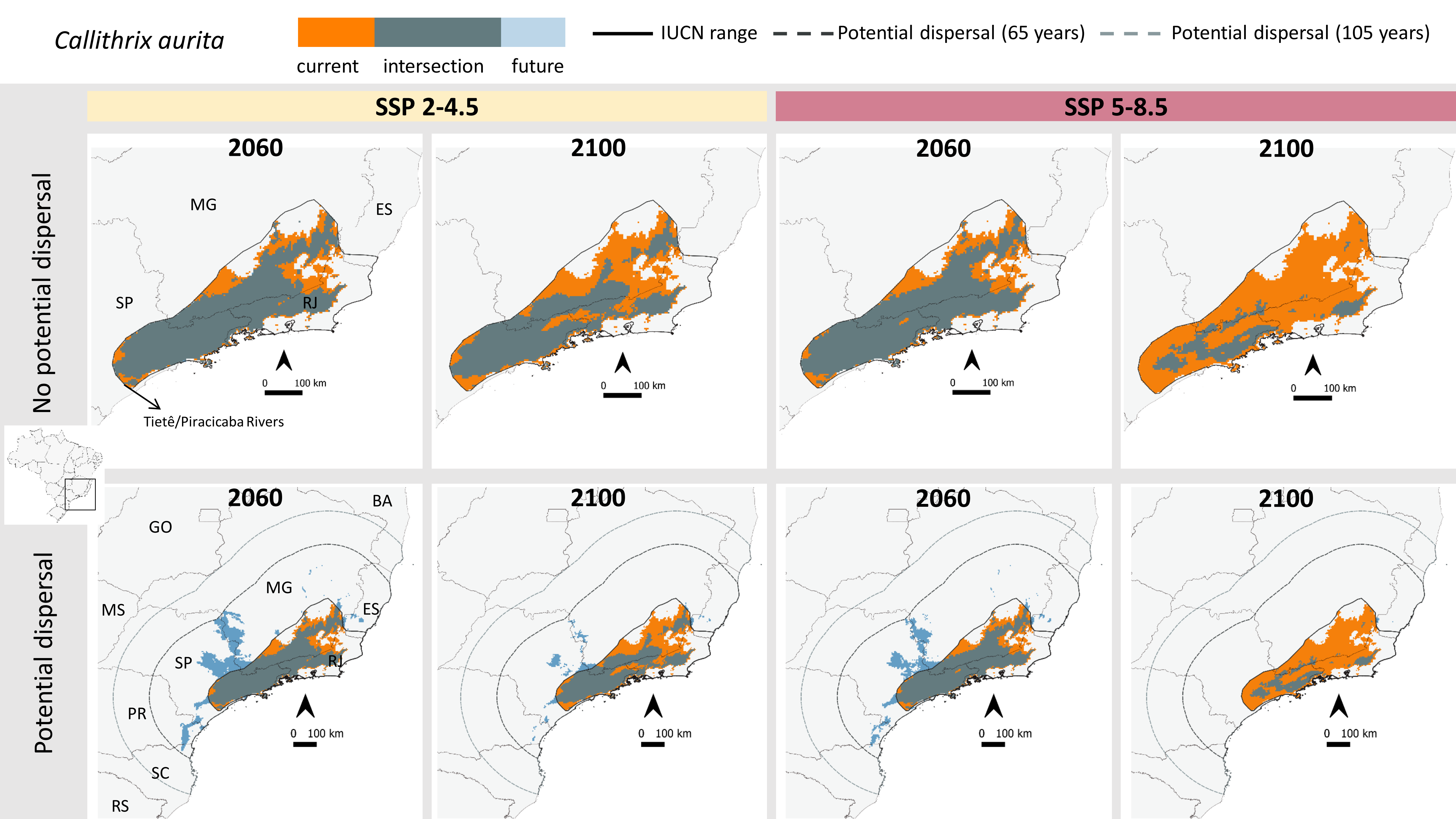

Callithrix aurita
current
intersection
future
IUCN range
Potential dispersal (65 years)
Potential dispersal (105 years)
SSP 2-4.5
SSP 5-8.5
No potential dispersal
Potential dispersal
2060
2100
MG
ES
SP
RJ
Tietê/Piracicaba Rivers
BA
GO
MS
PR
SC
RS
0 100 km

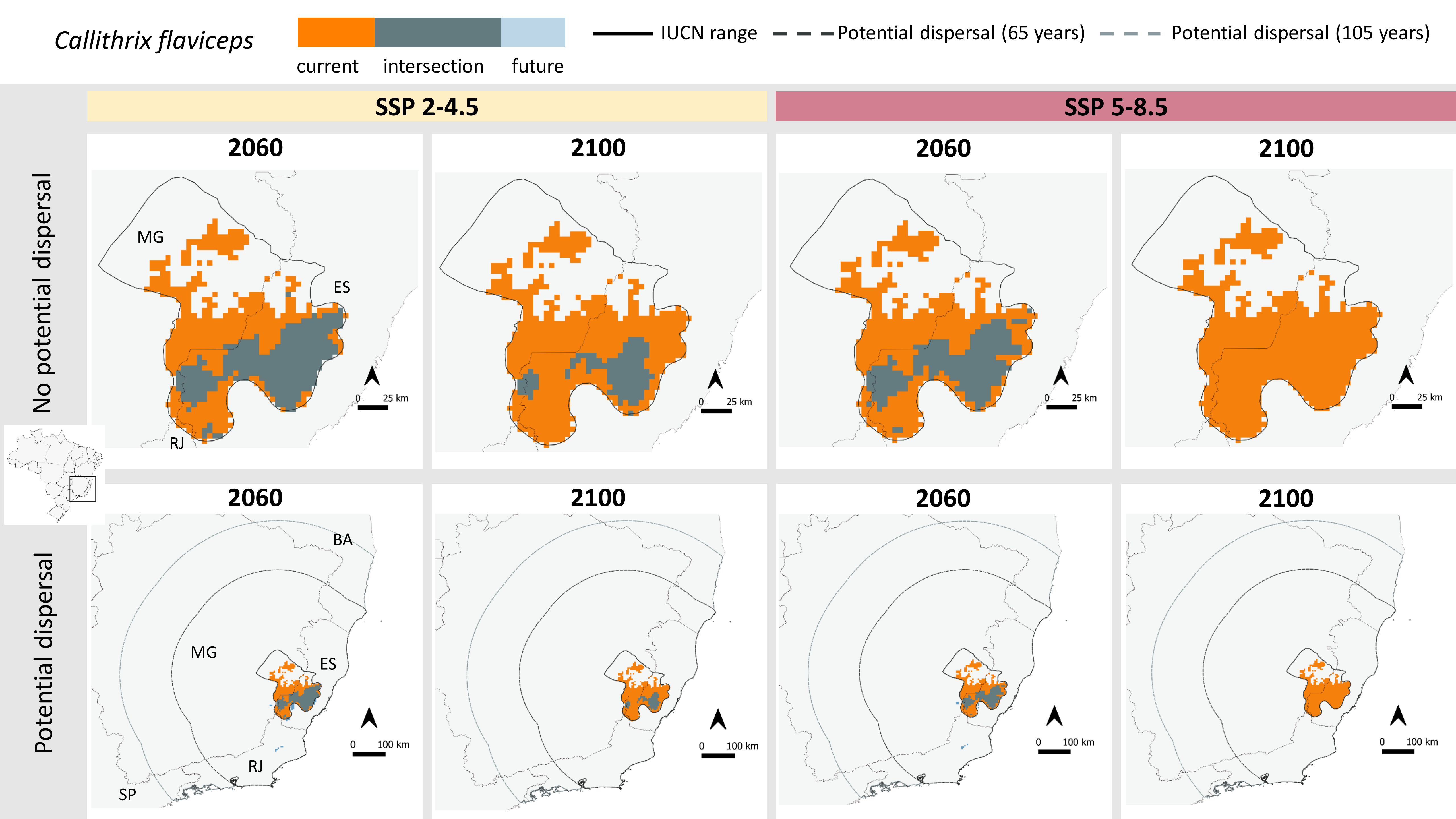

Callithrix flaviceps
current
intersection
future
IUCN range
Potential dispersal (65 years)
Potential dispersal (105 years)
SSP 2-4.5
SSP 5-8.5
2060
2100
2060
2100
No potential dispersal
Potential dispersal
MG
ES
RJ
BA
SP
0
25 km
100 km

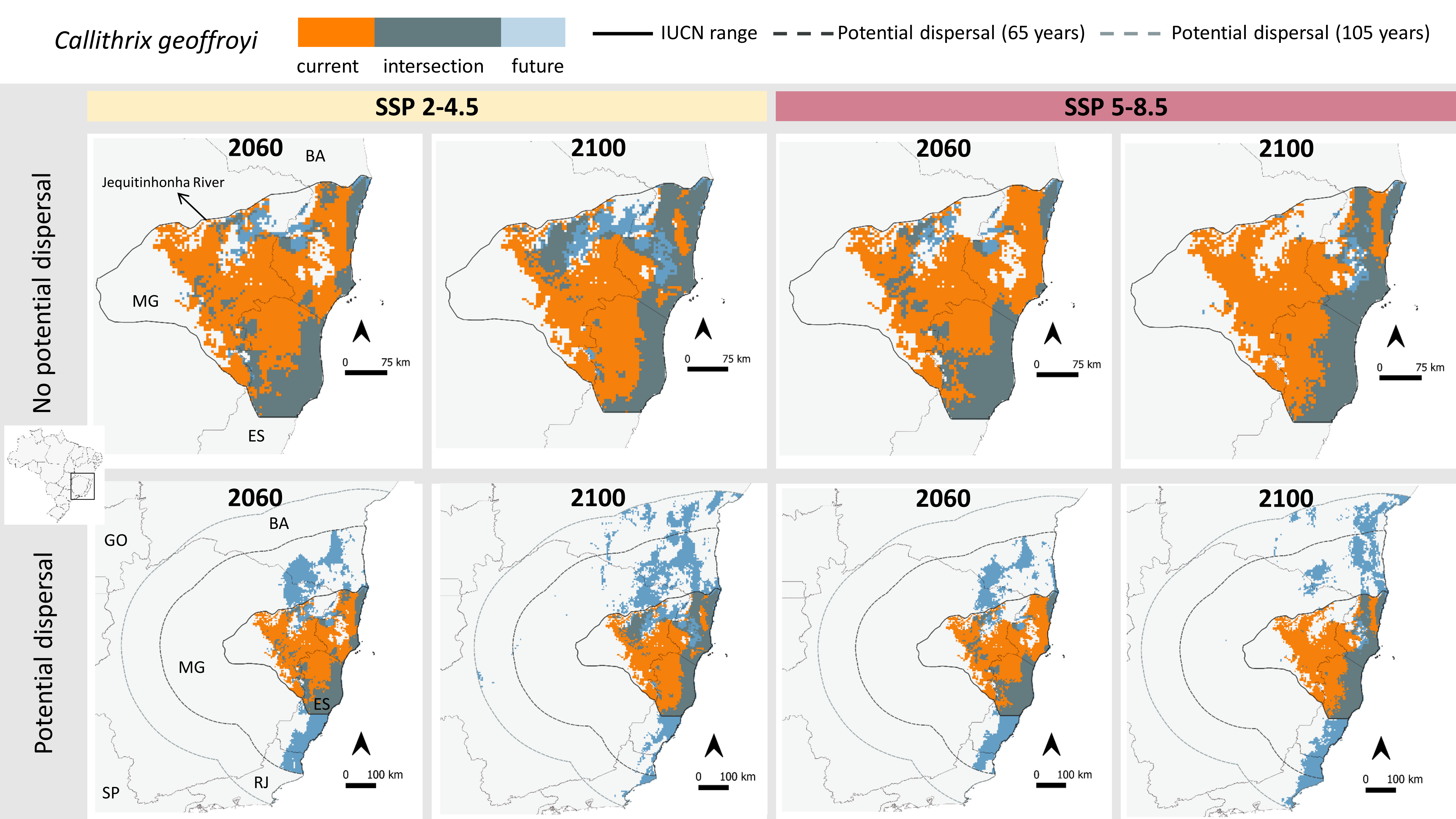

Callithrix geoffroyi
current
intersection
future
IUCN range
Potential dispersal (65 years)
Potential dispersal (105 years)
SSP 2-4.5
SSP 5-8.5
No potential dispersal
Potential dispersal
2060
2100
Jequitinhonha River
BA
MG
ES
GO
SP
RJ
0 75 km
0 100 km

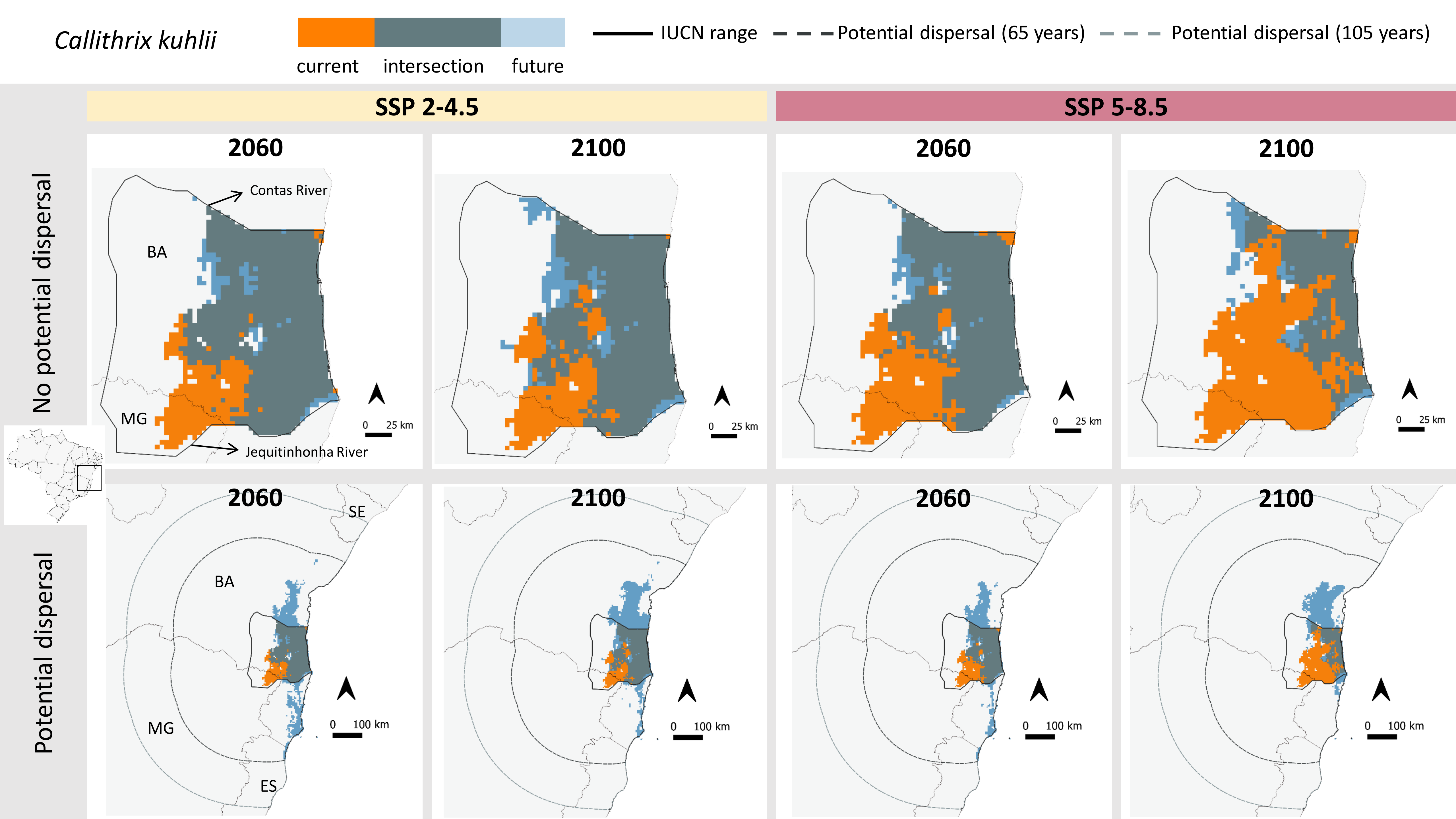
Callithrix kuhlii
current
intersection
future
IUCN range
Potential dispersal (65 years)
Potential dispersal (105 years)
SSP 2-4.5
SSP 5-8.5
2060
2100
2060
2100
No potential dispersal
Contas River
BA
MG
Jequitinhonha River
0 25 km
Potential dispersal
SE
BA
MG
ES
0 100 km

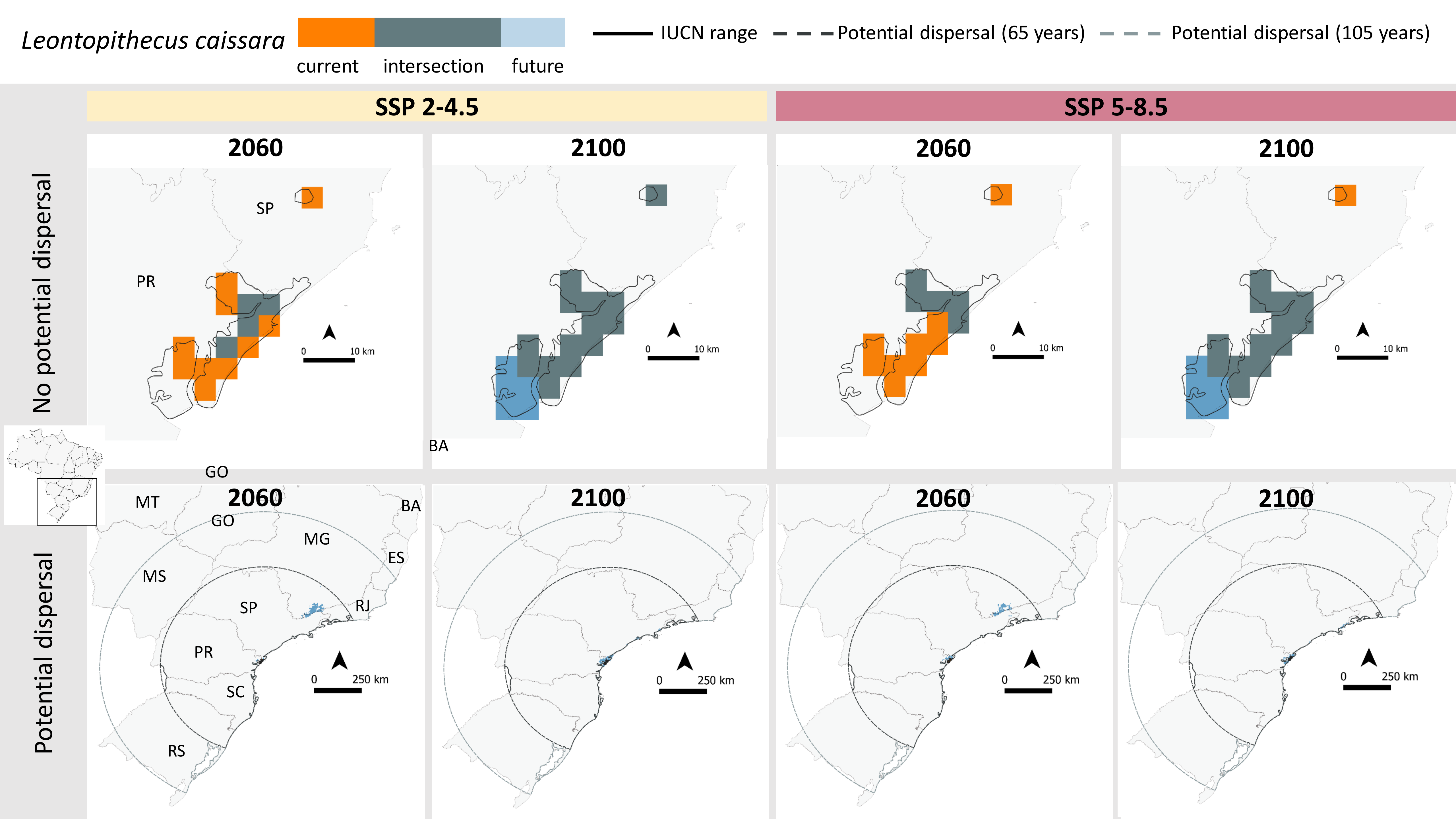

Leontopithecus caissara
current
intersection
future
IUCN range
Potential dispersal (65 years)
Potential dispersal (105 years)
SSP 2-4.5
SSP 5-8.5
2060
2100
2060
2100
No potential dispersal
Potential dispersal
SP
PR
BA
GO
MT
MG
ES
MS
RJ
SC
RS
0
10 km
0
250 km

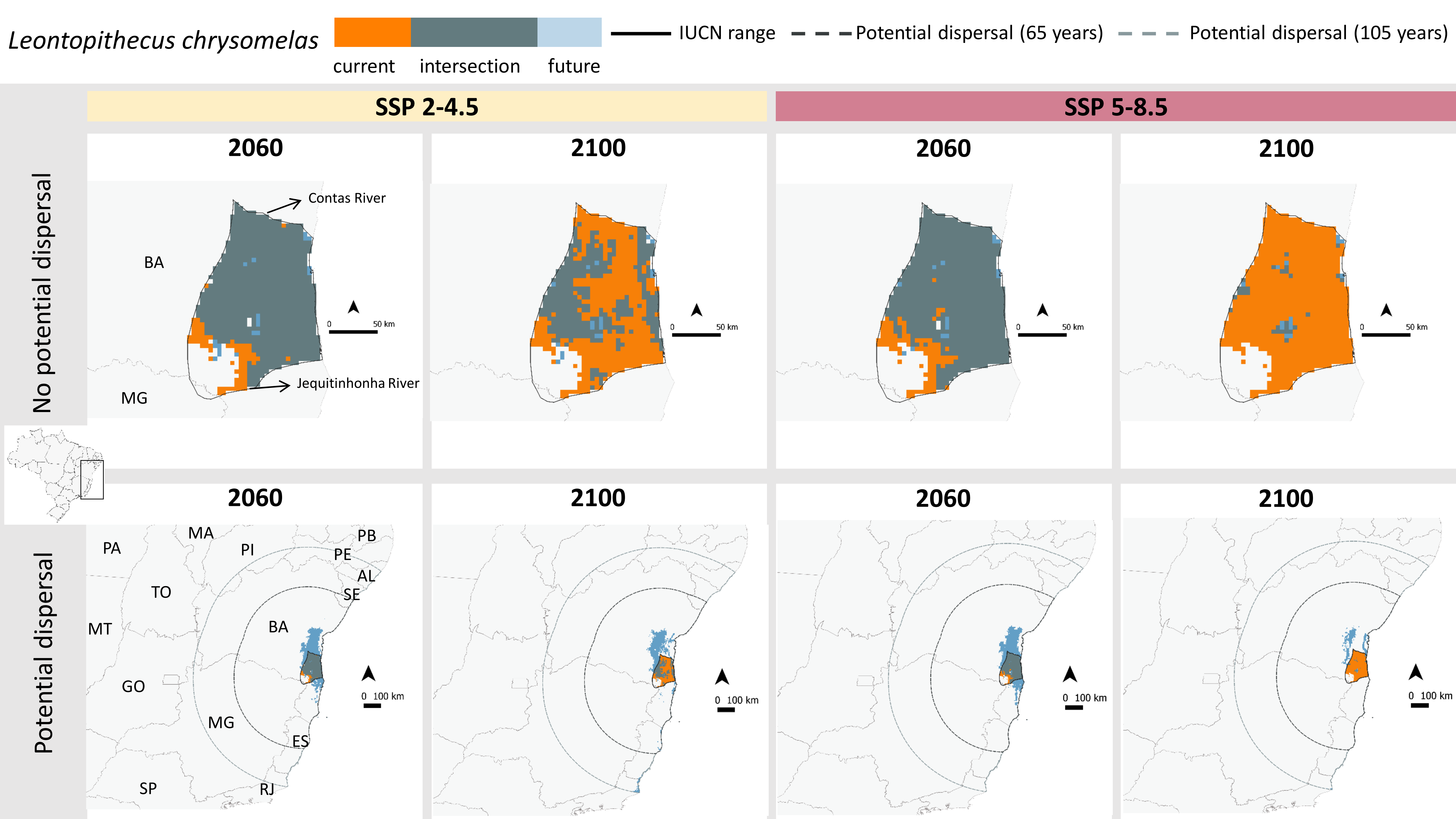

Leontopithecus chrysomelas
current
intersection
future
IUCN range
Potential dispersal (65 years)
Potential dispersal (105 years)
SSP 2-4.5
SSP 5-8.5
2060
2100
No potential dispersal
Potential dispersal
Contas River
Jequitinhonha River
BA
MG
0 50 km
0 100 km
PA
MA
PI
PB
PE
AL
SE
TO
MT
GO
ES
SP
RJ

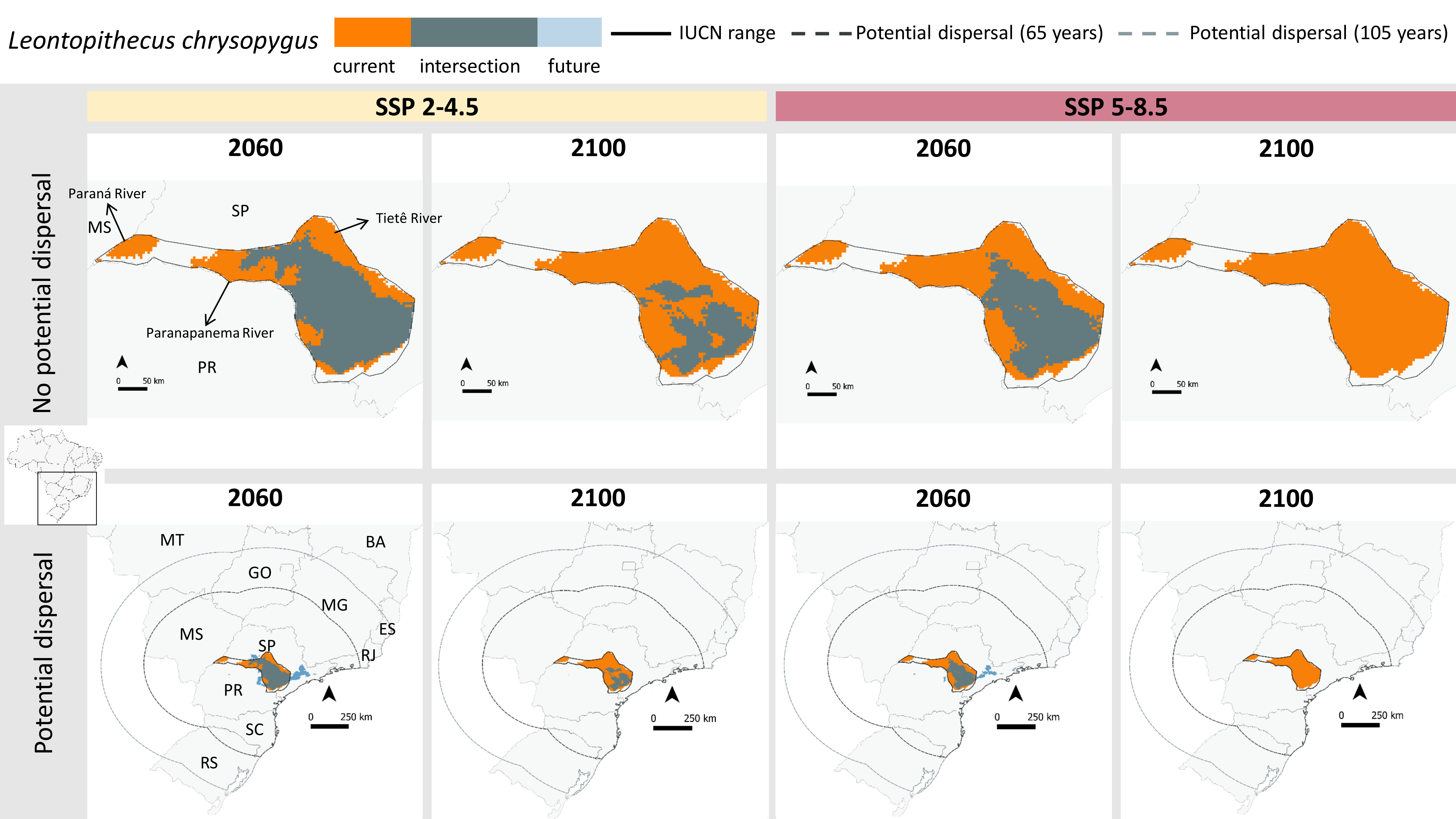

Leontopithecus chrysopygus
current
intersection
future
IUCN range
Potential dispersal (65 years)
Potential dispersal (105 years)
SSP 2-4.5
SSP 5-8.5
2060
2100
2060
2100
No potential dispersal
Paraná River
MS
SP
Tietê River
Paranapanema River
PR
0 50 km
Potential dispersal
MT
BA
GO
MG
ES
MS
SP
RJ
PR
SC
RS
0 250 km

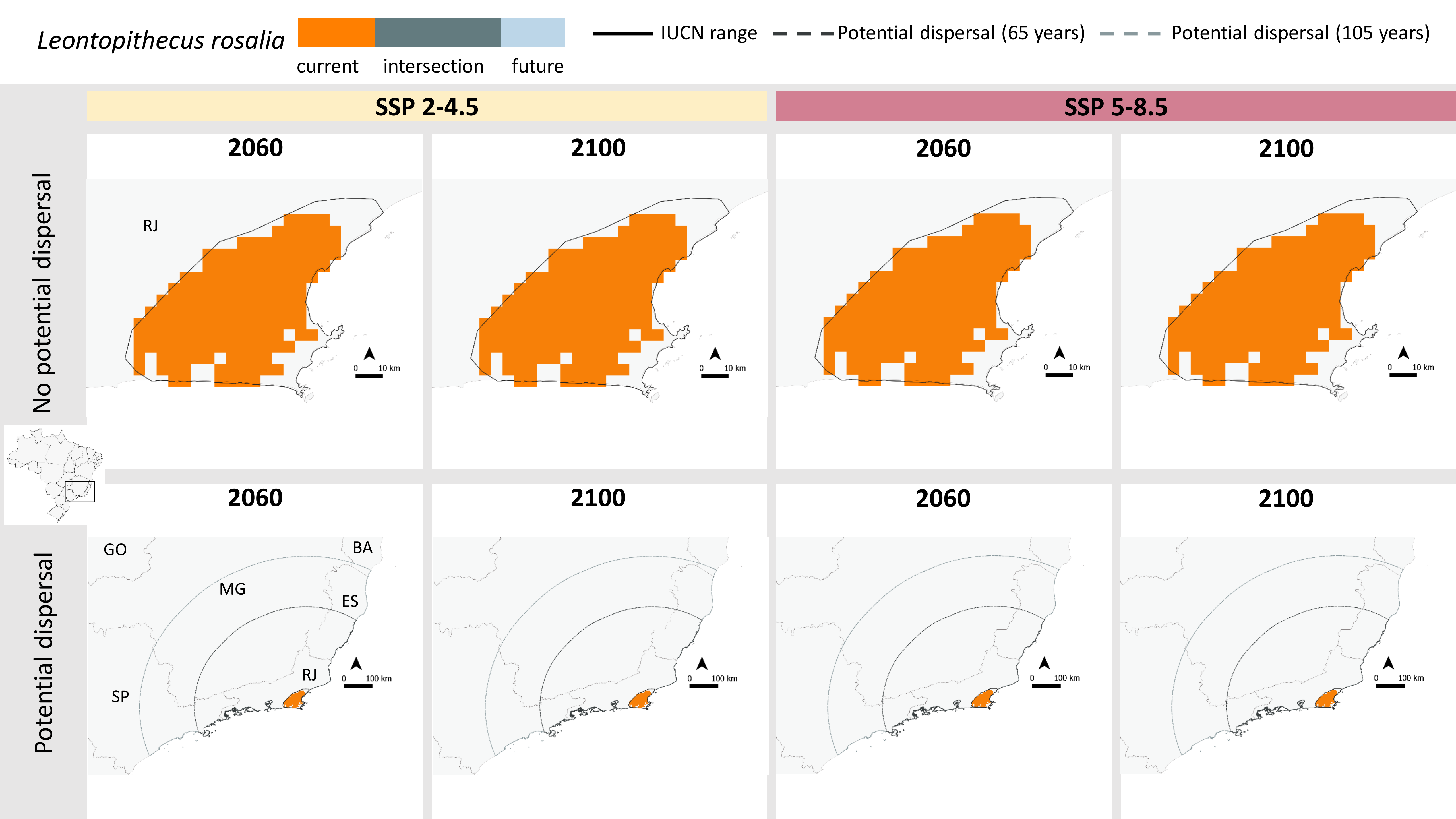

Leontopithecus rosalia
current
intersection
future
IUCN range
Potential dispersal (65 years)
Potential dispersal (105 years)
SSP 2-4.5
SSP 5-8.5
2060
2100
2060
2100
No potential dispersal
RJ
0
10 km
2060
2100
2060
2100
Potential dispersal
GO
BA
MG
ES
RJ
SP
0
100 km

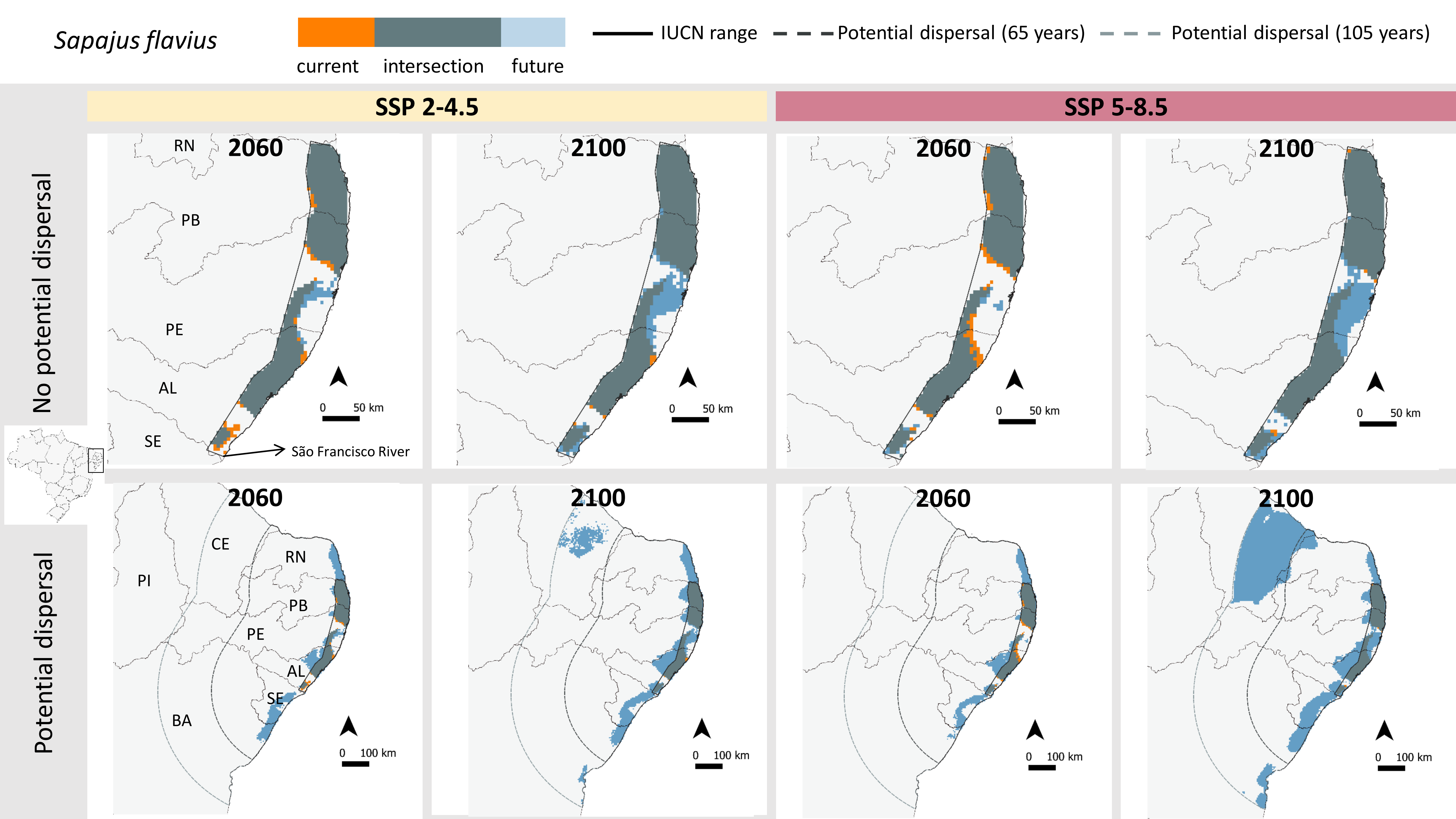
Sapajus flavius
current
intersection
future
IUCN range
Potential dispersal (65 years)
Potential dispersal (105 years)
SSP 2-4.5
SSP 5-8.5
No potential dispersal
Potential dispersal
2060
2100
RN
PB
PE
AL
SE
CE
PI
BA
São Francisco River
0 50 km
0 100 km

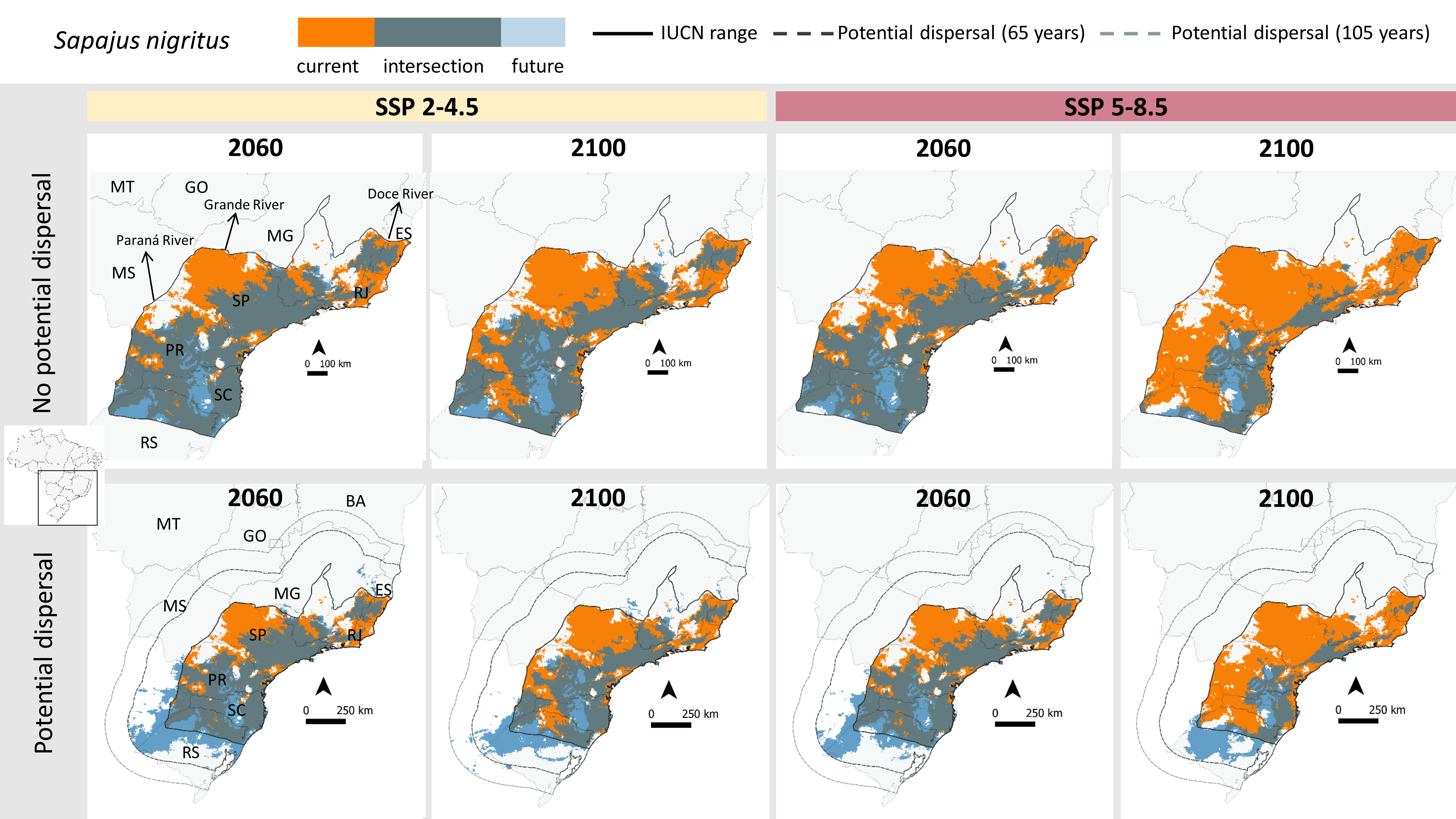

Sapajus nigritus
current
intersection
future
IUCN range
Potential dispersal (65 years)
Potential dispersal (105 years)
SSP 2-4.5
SSP 5-8.5
2060
2100
2060
2100
No potential dispersal
Potential dispersal
MT
GO
Grande River
Doce River
Paraná River
MG
ES
MS
SP
RJ
PR
SC
RS
BA
0 100 km
0 250 km

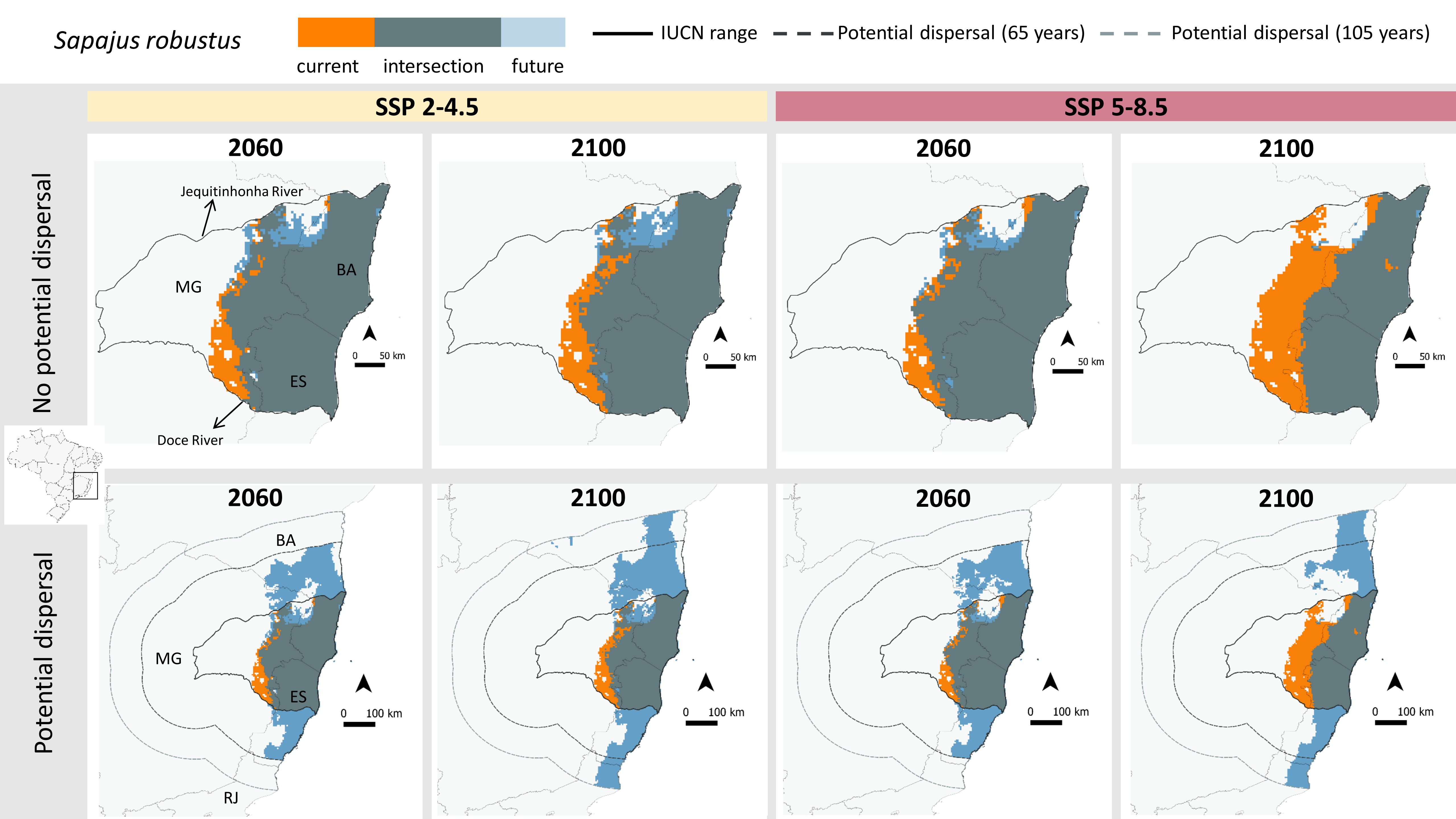
Sapajus robustus
current
intersection
future
IUCN range
Potential dispersal (65 years)
Potential dispersal (105 years)
SSP 2-4.5
SSP 5-8.5
No potential dispersal
Potential dispersal
2060
2100
2060
2100
Jequitinhonha River
Doce River
MG
BA
ES
RJ
0 50 km
0 100 km
